# Machine learning-based identification of glycosyltransferase-related mRNAs for improving outcomes and the anti-tumor therapeutic response of gliomas

**DOI:** 10.3389/fphar.2023.1200795

**Published:** 2023-08-16

**Authors:** Chunyu Zhang, Wei Zhou

**Affiliations:** ^1^ School of Medicine, Tongji University, Shanghai, China; ^2^ Department of Anesthesiology, Huzhou Central Hospital, The Affiliated Huzhou Hospital, Zhejiang University School of Medicine, Huzhou, Zhejiang, China

**Keywords:** glioma, glycosyltransferase, machine learning algorithms, prognosis, biomarker

## Abstract

**Background:** Glycosyltransferase participates in glycosylation modification, and glycosyltransferase alterations are involved in carcinogenesis, progression, and immune evasion, leading to poor outcomes. However, in-depth studies on the influence of glycosyltransferase on clinical outcomes and treatments are lacking.

**Methods:** The analysis of differentially expressed genes was performed using the Gene Expression Profiling Interactive Analysis 2 database. A total of 10 machine learning algorithms were introduced, namely, random survival forest, elastic network, least absolute shrinkage and selection operator, Ridge, stepwise Cox, CoxBoost, partial least squares regression for Cox, supervised principal components, generalized boosted regression modeling, and survival support vector machine. Gene Set Enrichment Analysis was performed to explore signaling pathways regulated by the signature. Cell-type identification by estimating relative subsets of RNA transcripts was used for estimating the fractions of immune cell types.

**Results:** Here, we analyzed the genomic and expressive alterations in glycosyltransferase-related genes in gliomas. A combination of 80 machine learning algorithms was introduced to establish the glycosyltransferase-related mRNA signature (GRMS) based on 2,030 glioma samples from The Cancer Genome Atlas Program, Chinese Glioma Genome Atlas, Rembrandt, Gravendeel, and Kamoun cohorts. The GRMS was identified as an independent hazardous factor for overall survival and exhibited stable and robust performance. Notably, gliomas in the high-GRMS subgroup exhibited abundant tumor-infiltrating lymphocytes and tumor mutation burden values, increased expressive levels of hepatitis A virus cellular receptor 2 and CD274, and improved progression-free survival when subjected to anti-tumor immunotherapy.

**Conclusion:** The GRMS may act as a powerful and promising biomarker for improving the clinical prognosis of glioma patients.

## Introduction

Glioma is the most frequently occurring type of brain cancer, and its most aggressive pathological form is known as brain glioblastoma (Louis et al., 2021). Gliomas have significant heterogeneity, and recent research has reported the tools for further classification of gliomas (Carlson et al., 2021; Rui et al., 2023). In clinical interventions, despite receiving the most aggressive treatments, glioblastoma patients’ median overall survival (OS) of less than 1.5 years is disheartening. Most antitumor drugs find it challenging to enter the brain due to the blood–brain barrier (BBB), a unique central nervous system (CNS) structure that poses a significant obstacle to developing anti-glioma treatments.

Despite the development of numerous anticancer drugs over the past decades, only few have been approved for the clinical treatment of gliomas (Oberoi et al., 2016; Nikolenko et al., 2020). For example, temozolomide (TMZ) mainly works by inducing DNA damage, thereby inhibiting DNA replication and cell proliferation (Lang et al., 2021). Significant advancements and progress have been made in immunotherapy for lung and liver cancers, resulting in notable enhancements in patient outcomes (Lee et al., 2015; Ferrarotto et al., 2021; Theelen et al., 2021). Unfortunately, however, only a minority of subjects respond to anti-glioma immunotherapeutic treatments, with most experiencing minimal or no clinical benefit. The response rate to immunotherapy for gliomas is disappointing, and regrettably, few patients receive clinical benefits and exhibit little or no improvement (Li C. et al., 2020). Exploring sophisticated immune contexts could reveal new therapeutic targets, as recent research has demonstrated that these factors influence the effectiveness of immunotherapy (Binnewies et al., 2018). The mounting evidence suggests that the immune reprogramming of glioma disrupts chemokines, cytokines, and growth factors in the glioma immune microenvironment (GIME), hindering the immune system’s ability to fight cancer (Klemm et al., 2020; Grabowski et al., 2021; Yu and Quail, 2021). The upregulation of immunosuppression-associated molecules, such as programmed cell death protein 1(PD1) and indoleamine 2,3-dioxygenase (IDO) in tumor cells, significantly impairs the immune system’s ability to recognize and attack cancer cells. These molecules act by inhibiting T-cell activation and promoting the development of regulatory T (Treg) cells, thus creating an immunosuppressive microenvironment that enables tumor cells to evade immune surveillance. The restricted antigen presentation mediated by PD1 and IDO further contributes to tumor immune escape by hampering effective antigen recognition by immune cells (Jiang et al., 2019). Remodeling of glioma-associated macrophages (GAMMs) promotes an increase in the production of immunosuppressive cytokines, such as interleukin-10 (IL-10) and transforming growth factor *ß* (TGF-β), relative to that in healthy donors (Coniglio and Segall, 2013), which supports the formation of a supportive microenvironment for an inefficient antitumor immune response (Garcia-Fabiani et al., 2020). The interaction between Treg cells and GAMMs in the GIME has a functional impact that leads to immunosuppression, promoting immune invasion and tumor progression. Treg cells play a specific role in inhibiting the activation and differentiation of CD4^+^ helper T cells and CD8^+^ cytotoxic T cells, thereby promoting the deficiency of antitumor immunity (Li C. et al., 2020). As a result, targeting these immunosuppressive pathways has become a promising strategy for enhancing antitumor immune responses and improving the clinical outcomes of cancer patients.

Glycosyltransferase is the enzyme responsible for catalyzing the formation of glycosidic linkages between or among substrates (Fuster and Esko, 2005). In recent years, cell-specific and tissue-specific aberrant glycan modifications associated with cancer have provided a strong basis for identifying potential targets in drug development and clinical management, thereby facilitating detection and treatment. The dysregulation of α2,6-sialylated lactosamine (Sia6LacNAc) has been identified as a prognosticator for adverse outcomes in colorectal cancer patients (Lise et al., 2000). The interaction of modified glycans on cancer cell surfaces with immune cells can lead to the activation of inhibitory immune processes, as widely documented (RodrÍguez et al., 2018). For example, the promotion of programmed cell death-ligand 1 (PD-L1) N-glycosyltransferase has been associated with *ß*-1,3-N-acetylglucosaminyl transferase (B3GNT3). In mouse breast cancer cells, the elimination of B3GNT3 decreases PD-L1 expression, ultimately amplifying the tumor rejection response (Pinho and Reis, 2015). An increase in T-cell receptor (TCR) signaling may possibly decrease the N-glycan branch of cytotoxic T-cell-associated antigen-4 (CTLA4), leading to the suppression of T-cell function and evasion of the immune system (Lau et al., 2007). The significant potential of glycosyltransferase-related markers for cancer management has been demonstrated by recent research, which found that the glycosyltransferase gene signature accurately predicts the prognosis of patients with pancreatic ductal adenocarcinoma (PDAC) (Mohamed Abd-El-Halim et al., 2021). The precise functions of these factors in glioma remain unclear and require further elucidation.

In this study, we examined 40 dysregulated glycosyltransferase-related genes that are associated with abnormal methylation and copy number variation in gliomas. The resulting glycosyltransferase-related mRNA signature (GRMS) was identified as an independent prognostic factor for gliomas and could serve as a potential tool for identifying patients who might benefit from chemotherapy or immunotherapy.

## Materials and methods

### Data sources and process

For glioma datasets from The Cancer Genome Atlas Program (TCGA), the Chinese Glioma Genome Atlas (CGGA), and the three sets from GlioVis databases, namely, Kamoun, Rembrandt, and Gravendeel, samples with complete follow-up information were included, and detailed clinical characteristics of the enrolled observations are displayed in Supplementary Table S1. In total, 2,030 glioma patients from five independent public datasets (TCGA-glioma, CGGA-glioma, Kamoun-glioma, Rembrandt-glioma, and Gravendeel-glioma) were included. The downloaded RNA-seq profiles (fragments per kilobase million (FPKM)-quantified values) were converted into transcripts per million (TPM)-quantified values and then subjected to log_2_ (TPM+1) normalization. The copy number variation (CNV) profile of gliomas, named “GBMLGG_minus_germline_cnv_hg19_seg.seg.txt,” was downloaded from the Broad GDAC Firehouse database (https://gdac.broadinstitute.org/) and analyzed by the genomic identification of significant targets in cancer, version 2.0 (GISTIC 2.0) module on the GenePattern website (https://cloud.genepattern.org/). DNA methylation data of gliomas were obtained from the UCSC Xena official website (https://xena.ucsc.edu/). The glioma somatic mutation data were collected by the R package TCGAbiolinks and analyzed by the R package maftools. The correlation of DNA methylation changes and copy number variations (CNVs) with the corresponding mRNA expression levels was calculated based on the Spearman correlation analysis.

### Gene selection and survival analysis

A glycosyltransferase gene group including 202 mRNAs was acquired based on the Hugo Gene Nomenclature Committee (https://www.genenames.org/data/genegroup/#!/group/424). The differentially expressed genes (DEGs) between low-grade gliomas (LGGs) and non-tumor samples, as well as DEGs between glioblastomas (GBMs) and non-tumor samples, with the cutoffs of log_2|_fold change (FC)|>1 and false discovery rate (FDR) < 0.05, were downloaded from the Gene Expression Profiling Interactive Analysis 2 (GEPIA2). Finally, 40 glycosyltransferase-associated genes were obtained (Supplementary Table S2). To establish a consensus GRMS with significant prediction accuracy, we introduced 80 algorithm combinations based on 10 machine learning algorithms. The 10 methods included random survival forest (RSF), elastic network (Enet), least absolute shrinkage and selection operator (Lasso), Ridge, stepwise Cox, CoxBoost, partial least squares regression for Cox (plsRcox), supervised principal components (SuperPC), generalized boosted regression modeling (GBM), and survival support vector machine (survival-SVM). The GRMS-generated process was described as follows: 1) univariate Cox regression analysis was initially applied to explore prognosis-associated mRNAs in the CGGA glioma cohort; 2) then, 80 combined algorithms were carried out on the candidate mRNAs to fit predictive models based on the leave-one-out cross-validation (LOOCV) framework in the CGGA cohort; 3) all models were validated in five glioma datasets (TCGA-Glioma, Kamoun, Rembrandt, Gravendeel, and the merged five cohorts); and 4) for each model, the concordance index (C-index) was computed, and the model with the highest average C-index was regarded optimally and sent to the subsequent analysis. High- and low-GRMS subgroups were identified based on the surv_cutpoint function in the R package survminer. Kaplan–Meier (K–M) curves were generated, and the OS difference was calculated by the log-rank test. We further assessed the capability of the GRMS to predict the prognosis of glioma samples using receiver operating characteristic (ROC) curves.

### Enrichment analysis

The empirical Bayesian method in the R package limma was introduced (Ritchie et al., 2015) to calculate the log_2_|FC| between the divided groups in the six cohorts. Gene Set Enrichment Analysis (GSEA) was performed based on R package clusterProfiler (Yu et al., 2012) on the log_2_|FC| value-ranked genes. In addition, the document “h.all.v7.4. symbols.gmt” was selected as the reference.

### Evaluation of tumor microenvironment immunological characteristics

We collected 69 immunomodulators, such as the major histocompatibility complex (MHC), receptors, chemokines, immunostimulants, and inhibitors, from previous research (Thorsson et al., 2018; Chen et al., 2022; Qing et al., 2022; Zhang et al., 2022). The correlation of the mRNA expression of immunomodulators with GRMS values was calculated based on the Spearman correlation analysis. For further insights into the association of the GRMS with immune infiltrates in the tumor microenvironment (TME) of gliomas, we introduced two methods, including the single-sample gene set enrichment analysis (ssGSEA) and Microenvironment Cell Populations-counter (MCP-counter) algorithms (Barbie et al., 2009; Becht et al., 2016). Moreover, the Estimation of STromal and Immune cells in MAlignant Tumor tissues using Expression data (ESTIMATE) method, an indicator for the levels of tumor-infiltrating immune cells, was used to calculate the immune score for each glioma sample (Yoshihara et al., 2013).

### Genomic alterations between GRMS subgroups

The R package ‘maftools’ was introduced to generate a waterfall chart of gene mutations in the TCGA glioma cohort. For analysis of the copy number, CNVs were analyzed with GISTIC 2.0 on the GenePattern website (https://cloud.genepattern.org/), including copy number gain and deletion, and the human hg19 genome sequence was used as a reference set (Mermel et al., 2011). For analyzing CNVs on the GISTIC 2.0 website, the default parameters were selected.

### Correlation of the GRMS with anti-tumor therapy

To estimate the potential of the GRMS in clinical applications, we first used the subclass mapping method (SubMap)-embedded data and human immunotherapy transcriptome data to further explore the predictive ability of GRMS in anti-tumor immunotherapy (Lu et al., 2019; Hoshida et al., 2007). In addition, we downloaded the drug information from the public database, Genomics of Drug Sensitivity in Cancer (GDSC, https://www.cancerrxgene.org/). R package pRRophetic was applied to assess the sensitivity of patients in high- and low-GRMS subgroups to chemotherapeutic agents.

### Statistical analysis

All the statistical analyses were conducted in the R project (v4.0.5). The Shapiro–Wilk test was used to examine the normal distribution of the continuous variables. In order to assess the correlation between two continuous variables, the Spearman correlation coefficient was used. The continuous variables were compared using the Mann–Whitney test or *t*-test. The R package ‘survival’ was introduced to perform Cox regression analysis and Kaplan–Meier analysis. The time-dependent area under the ROC curve (AUC) analysis was conducted by the “timeROC” R package. The DEGs between high- and low-GRMS subgroups were identified by the “Limma” R package, and genes with |log_2_FC| and FDR<0.05 were considered to be expression-changed. All tests were two-sided, and *p* < 0.05 was considered significant.

## Results

### Genetic characteristics and transcriptional variations of glycosyltransferase-related genes

**FIGURE 1:**
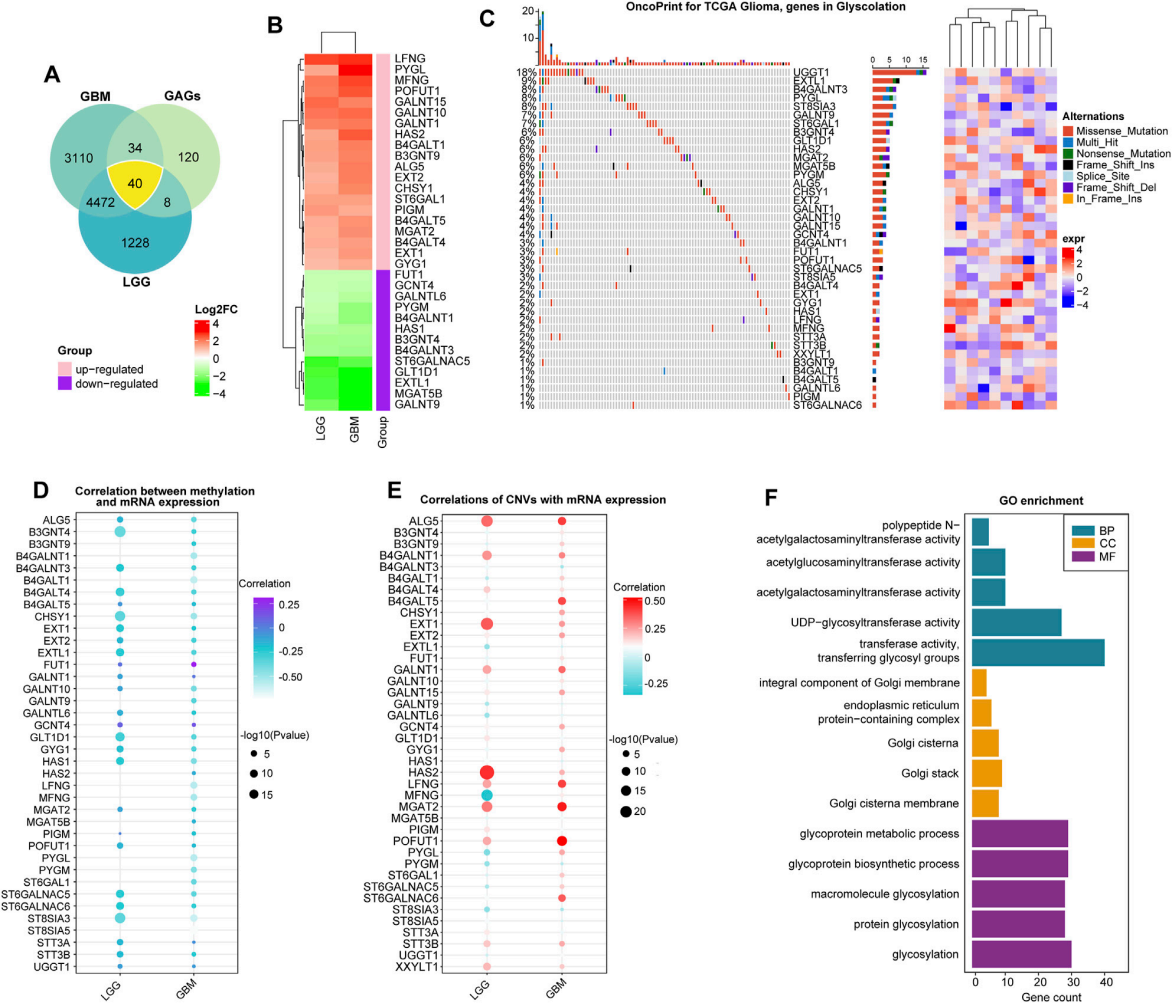
Expression alterations of glycosyltransferase-related molecules. **(A)** The Venn diagram demonstrates that 40 glycosyltransferase-associated genes had changed expression in gliomas, including LGGs and GBMs, compared with non-tumor controls. **(B)** Heatmap of log_2_FC values of the DEGs in LGGs and GBMs. In LGGs and GBMs, the red color represents overexpressed genes, and the green color represents lowly expressed genes. **(C)** The waterfall plot demonstrates the somatic mutation profiles of the mutated glycosyltransferase molecules in the TCGA-glioma cohort. **(D)** The bubble chart demonstrates the correlation between methylation data and the mRNA expression of the 40 DEGs. The purple color represents a positive correlation, and the blue color shows a negative correlation. **(E)** The bubble chart reveals the correlation between the CNV and mRNA expression of the 40 DEGs. The red color indicates a positive correlation, and the blue color indicates a negative correlation. **(F)** Gene Ontology analysis of 40 DEGs in terms of biological processes (BPs), cellular components (CCs), and molecular functions (MFs).

We summarized data on glycosyltransferase-related molecules in glioma samples and non-tumor brain tissues using the GEPIA2 website (http://gepia2.cancer-pku.cn/) (Figure 1A). As a result, 16 DEGs were overexpressed, while 24 DEGs were downregulated in glioma tissues, in comparison to the expression data of DEGs in non-tumor brain samples (Figure 1B and Supplementary Table S2). Then, we obtained somatic mutation files of the TCGA-glioma cohort based on the TCGAbiolinks package (Colaprico et al., 2016). We showed the somatic mutations of 40 glycosyltransferase molecules using a waterfall diagram, and UGGT1 had the highest mutation percentage (18%) (Figure 1C). The gene methylation profiles were compared between glioma and normal tissues, and 24 genes exhibited dysregulated methylation. In detail, nine molecules were hypermethylated in gliomas: UDP-GlcNAc:betaGal beta-1,3-N-acetylglucosaminyltransferase 4 (B3GNT4), beta-1,4-galactosyltransferase 1 (B4GALT1), B4GALT4, polypeptide N-acetylgalactosaminyltransferase 10 (GALNT10), glucosaminyl (N-acetyl) transferase 4 (GCNT4), glycosyltransferase 1 domain containing 1 (GLT1D1), alpha-1,6-mannosylglycoprotein 6-beta-N-acetylglucosaminyltransferase B (MGAT5B), xyloside xylosyltransferase 1 (XXYLT1), and ST8 alpha-N-acetyl-neuraminide alpha-2,8-sialyltransferase 5 (ST8SIA5), and 15 molecules were hypomethylated in gliomas: UDP-glucose glycoprotein glucosyltransferase 1 (UGGT1), STT3 oligosaccharyltransferase complex catalytic subunit B (STT3B), STT3A, ST6 N-acetylgalactosaminide alpha-2,6-sialyltransferase 6 (ST6GALNAC6), glycogen phosphorylase L (PYGL), protein O-fucosyltransferase 1 (POFUT1), phosphatidylinositol glycan anchor biosynthesis class M (PIGM), alpha-1,6-mannosyl-glycoprotein 2-beta-N-acetylglucosaminyltransferase (MGAT2), hyaluronan synthase 2 (HAS2), GALNT15, exostosin glycosyltransferase 2 (EXT2), B4GALT5, beta-1,4-N-acetyl-galactosaminyltransferase 1 (B4GALNT1), chondroitin sulfate synthase 1 (CHSY1), and ALG5 dolichyl-phosphate beta-glucosyltransferase (ALG5) (*p* < 0.05, Supplementary Figures S1, S1B). In total, 11 genes demonstrated consistent negative correlations with the corresponding mRNA in LGGs and GBMs: B4GALNT1, B4GALT1, CHSY1, GALNT9, LFNG O-fucosylpeptide 3-beta-N-acetylglucosaminyltransferase (LFNG), MFNG O-fucosylpeptide 3-beta-N-acetylglucosaminyltransferase (MFNG), PYGL, glycogen phosphorylase, muscle associated (PYGM), ST6 beta-galactoside alpha-2,6-sialyltransferase 1 (ST6GAL1), ST8 alpha-N-acetyl-neuraminide alpha-2,8-sialyltransferase 3 (ST8SIA3), and ST8SIA5 (|cor|>0.3, *p* < 0.05, Figure 1D and Supplementary Table S3). In LGG samples, the expression levels of B3GNT4, B3GNT9, HAS2, and MGAT5B were negatively correlated with those of the relevant mRNAs, and in GBMs, the expression levels of ALG5, B4GALT4, EXT2, exostosin like glycosyltransferase 1 (EXTL1), FUT1, GALNT10, GLT1D1, glycogenin 1 (GYG1), HAS1, and ST6 N-acetylgalactosaminide alpha-2,6-sialyltransferase 5 (ST6GALNAC5) were uniquely negatively associated with their mRNA expression levels (|cor|>0.3, *p* < 0.05, Figure 1D, and Supplementary Table S3). Finally, we confirmed that genetic variations could affect the expressive features of glycosyltransferase-associated DEGs. Analysis showed that the most common gene alteration types for glycosyltransferase-related genes were highly heterogeneous amplification and heterozygous deletion in glioma samples. For example, five genes in LGG and seven genes in GBMs demonstrated a close correlation between the CNV and mRNA levels in gliomas (|cor|>0.3, *p* < 0.05, Figure 1E, Supplementary Table S4, Supplementary Figures S2A, 2B), demonstrating that the expression dysregulation of DEGs was significantly correlated with genome alterations. Enrichment analysis on 40 DEGs demonstrated that, in GO analysis, DEGs were involved in protein glycosyltransferase, UDP-glycosyltransferase activity, acetylgalactosaminyltransferase activity, and the integral component of the Golgi membrane (Figure 1F and Supplementary Table S5).

### Relationship between glycosyltransferase-related regulators and cancer pathways

**FIGURE 2:**
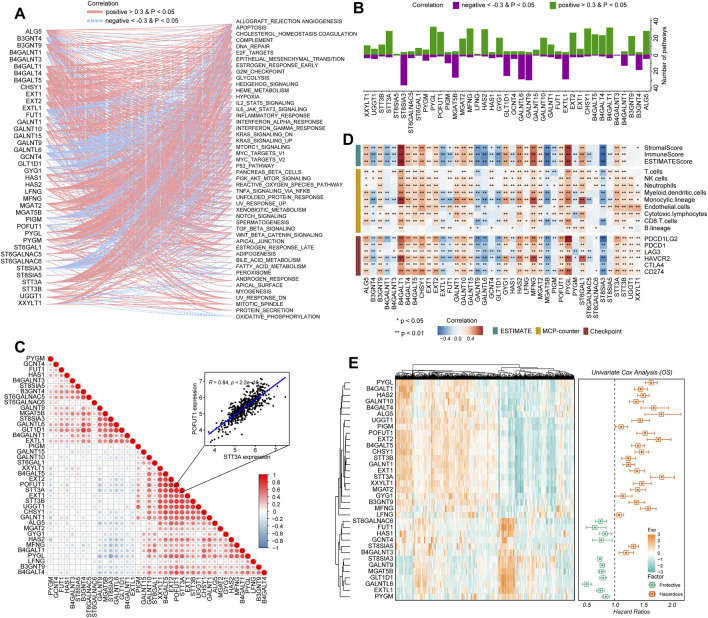
Immuno-oncological features and clinical significance of glycosyltransferase regulators in gliomas. **(A)** Network diagram displays the relationship between the selected glycosyltransferase regulators and cancer hallmark-related pathways in gliomas. **(B)** The bar plot demonstrates the number of the pathways positively and negatively correlated with glycosyltransferase regulators. **(C)** The heatmap demonstrates the correlation of glycosyltransferase regulators with tumor purity, immune score, and stromal score in gliomas (upper), with immune infiltrates (middle), and with the expression levels of immune checkpoints (lower). **(D)** Correlations between the expressions of each glycosyltransferase regulator in gliomas. The red color indicates a positive correlation, and the blue color indicates a negative correlation. **(E)** Univariate Cox regression of the prognostic role of glycosyltransferase regulators in the CGGA set. The heatmap demonstrates the scaled expression profiles of genes in gliomas (left), and the forest plot reveals the HR and the corresponding 95% CI of the included genes.

To determine how glycosyltransferase-related regulators were involved in glioma pathophysiology, a correlation degree was calculated between glycosyltransferase-involved regulators and 50 tumor hallmark-associated signaling pathways. The expression levels of regulators were strongly correlated with statuses of multiple oncogenesis-associated pathways. With the higher number of connections with activated pathways, including epithelial–mesenchymal transition (EMT) and the phosphoinositide 3-kinase (PI3K)/AKT serine/threonine kinase 1 (AKT)/mechanistic target of the rapamycin kinase (mTOR) pathway, PYGL, MFNG, CHSY1, STT3A, HAS2, and B4GALT4 demonstrated a sophisticated association with tumor promotion in gliomas (Figures 2A, B). However, EXTL1, ST8SIA3, and GALNT9 demonstrated a negative relation with oncogenic pathways, suggesting a potentially protective role for glioma progression (Figures 2A, B). Then, the correlation analysis of genes with 10 cancer-related pathways at the protein level was introduced by the online tool-GSCA (Liu et al., 2022). We found that, for example, B4GALT4, PYGL, and STT3A were strongly correlated with the high level of EMT activity (percent: 100%); STT3A and CHSY1 were related to DNA damage activation; MFNG, HAS2, and CHSY1 were associated with apoptosis activation; and EXTL1 was related to the inhibition of cell cycle activity. In addition, ST8SIA3 and GALNT9 might have an inhibitive role in cancer promotion by negatively modulating the activity of DNA damage and the EMT, PI3K/AKT, and mTOR pathways (Supplementary Figure S3). Notably, a growing body of evidence suggests that the collaboration of aberrantly glycosylate-related modulators influences cancer progression and development (Thomas et al., 2021). Therefore, the correlation between these 40 regulators in gliomas was examined. These associations showed that the interactions among the candidates might influence glioma development. In detail, STT3A and POFUT1 demonstrated the highest correlation degree (Figure 2C, R = 0.84), which might demonstrate the synergistic effects of both genes to promote glioma malignant behaviors. The ESTIMATE and MCP-counter algorithms were introduced to detect the relationship of stromal and immune cells with glycosyltransferase-associated modulators in expression levels (Figure 2D). Frequent positive correlations were identified between XXYLT1, UGGT1, STT3B, STT3A, ST6GAL1, PYGL, POFUT1, MGAT2, MFNG, LFNG, HAS2, GYG1, GALNT15, GALNT10, GALNT1, EXT2, EXT1, CHSY1, B4GALT5, B4GALT4, B4GALT1, B3GNT9, ALG5, and immune-stromal cells, as well as immune checkpoints. In contrast, ST8SIA3, ST6GALNAC5, PYGM, MGAT5B, HAS1, GLT1D1, GCNT4, GALNTL6, GALNT9, FUT1, EXTL1, B4GALNT3, B4GALNT1, and B3GNT4 had a negative correlation with immune-stromal cells and immune checkpoints (Figure 2D). Such findings indicated that different glycosyltransferase modification patterns were closely associated with anti-tumor and pro-tumor immunity.

### Construction of the GRMS model

**FIGURE 3:**
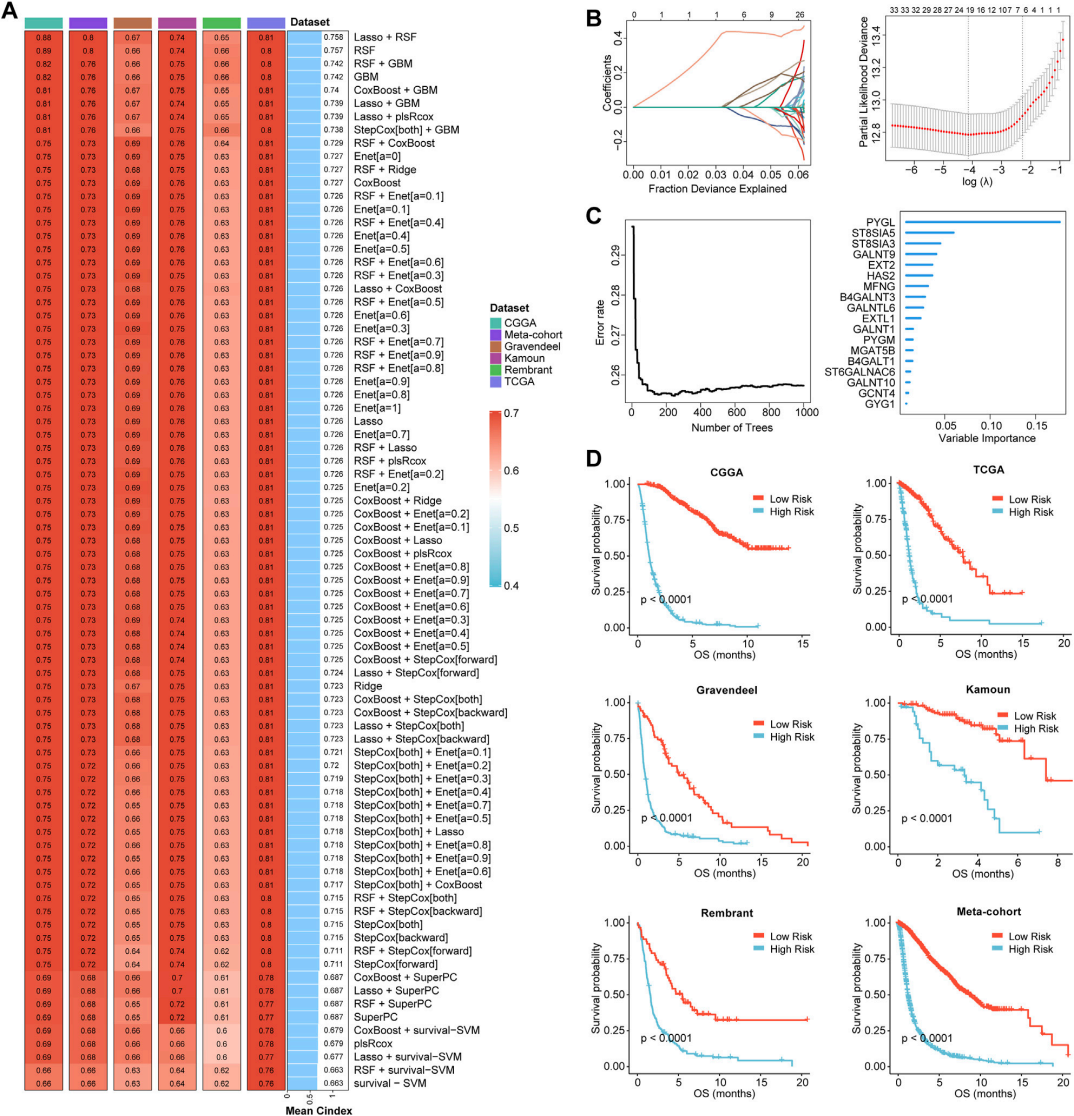
Prognostic significance of the GRMS. **(A)** The complex heatmap demonstrates a total of 80 kinds of prediction models via the LOOCV framework and further calculated the C-index of each model across all the six datasets. The heatmap displays the C-index values (left). Rows represent the algorithms, and columns represent the dataset. The bar plot demonstrates the average C-index value of the six sets. **(B)** In the CGGA cohort, optimal *λ* was determined when the partial likelihood deviance reached the minimum value and further generated Lasso coefficients of the most useful prognostic genes. **(C)** The number of trees for determining the GRMS with a minimal error and the importance of the 18 most valuable mRNAs based on the RSF algorithm. **(D)** K–M curves demonstrate a significant difference in the survival rate between the high- or low-GRMS subgroups.

**FIGURE 4:**
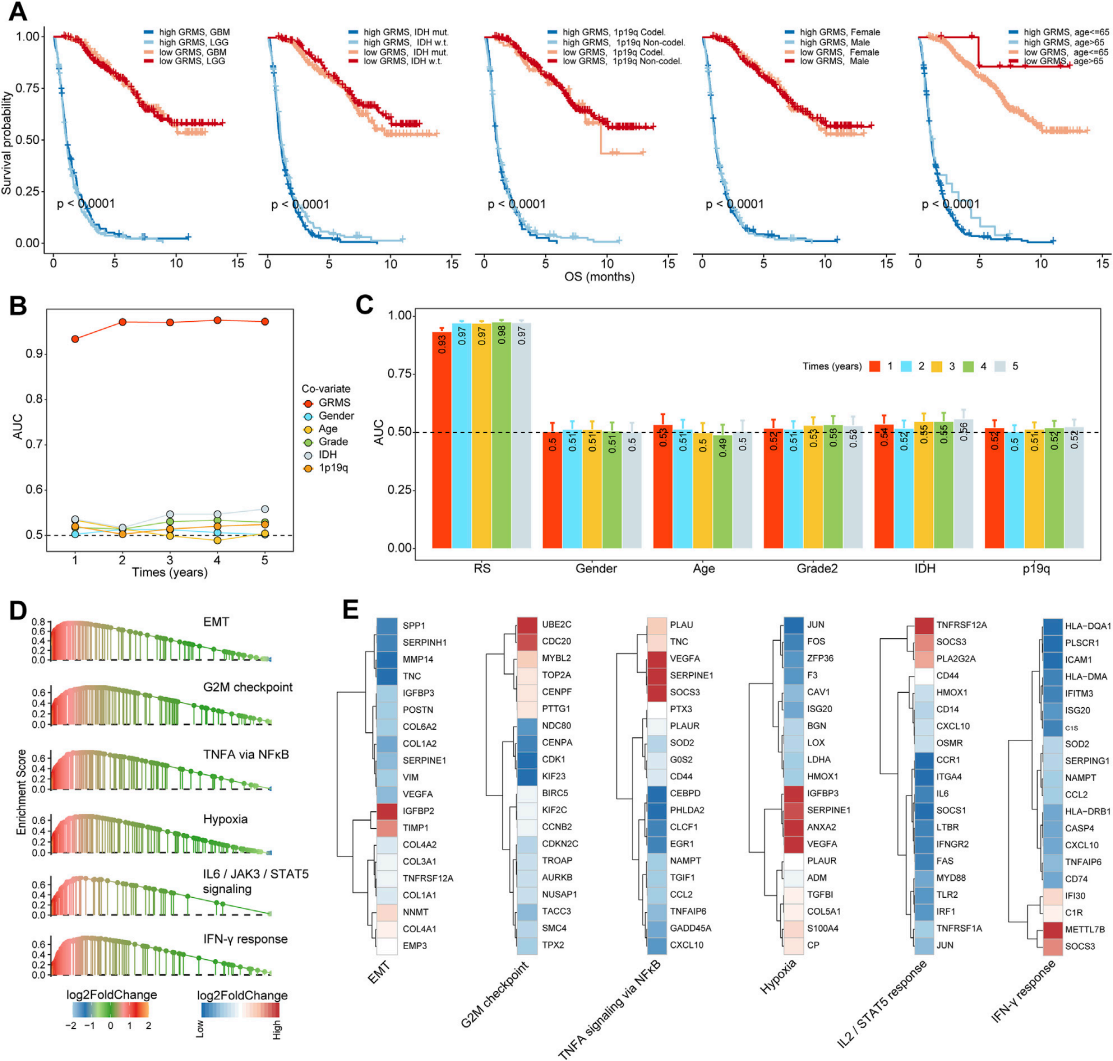
Stratification survival analysis of the GRMS in gliomas. **(A)** K–M curves of gliomas in the CGGA cohort based on the combined effects of the GRMS with WHO grade, IDH status, 1p19q status, gender, and age on prognosis. **(B, C)** Time-dependent AUC values of the GRMS at 1-, 2-, 3-, 4-, and 5-year OS in the CGGA dataset. **(D)** GSEA plot reveals the upregulated pathways related to the GRMS. The red color represents the positive value of log_2_FC of each gene, and the green color represents the negative value of log_2_FC of each gene. **(E)** Heatmaps of log_2_FC values of top 20 DEGs between the GRMS subgroups involved in the corresponding pathways. The red color represents the positive value of log_2_FC of each gene, and the blue color represents the negative value of log_2_FC of each gene.

Based on the expression profiles of 40 glioma-related mRNAs, univariate Cox analysis filtered out 34 DEGs, which were associated with glioma prognosis in the CGGA glioma cohort (*p* < 0.05, Figure 2E). A consensus GRMS was developed using the machine learning-based integrative framework at the root of the prognostic 34 mRNAs. In the CGGA-glioma dataset, we fitted 80 kinds of prediction models via the LOOCV framework and further calculated the C-index of each model across all validation datasets (Figure 3A; Supplementary Table S6). Interestingly, the optimal model was computed by the Lasso and random forest with the highest average C-index (0.758), and this model had a leading C-index among all validation datasets (Figure 3A). In the Lasso regression analysis, 34 mRNAs were filtered out (Figure 3B), and 18 candidates were subjected to the identification of the GRMS (Figure 3C). Next, the risk score-GRMS for each patient with glioma was computed based on the expression of 18 candidate genes by the rfsrc function in the randomForestSRC package. Stratification of glioma samples into two groups was achieved by employing the optimal GRMS cutoff function in the R package ‘survminer’. As illustrated in Figure 3D, based on K–M plots, samples with high GRMS had significantly shorter OS compared to those with low GRMS (*p* < 0.05). Previous studies proposed that clinical characteristics (e.g., WHO grade) and molecular alterations (e.g., IDH mutation) could influence the prognosis of gliomas. K–M plots demonstrated that the clinical outcomes of samples with different clinicopathological characteristics could still be separated according to the GRMS (*p* < 0.001, Figure 4A and S4A-E), which demonstrated that the potential marker might provide significant OS stratification for gliomas. Multivariate Cox regression indicated that the GRMS remained a risk factor (hazard ratio (HR) > 1.00) with statistical significance (*p* < 0.05) after adjusted by available clinical and pathological features of gliomas, such as age, gender, histology, IDH mutation status, and chromosome 1p19q codeletion status, which suggested that the GRMS could be identified and validated as an independent risk factor for OS of glioma patients (Supplementary Figures S5A–F)). ROC analysis measured the discriminative ability of the GRMS, with 1-, 2-, 3-, 4-, and 5-year AUCs of 0.934, 0.971, 0.970, 0.975, and 0.972 in the CGGA dataset; 0.836, 0.772, 0.743, 0.752, and 0.691 in the Kamoun dataset; 0.740, 0.787, 0.832, 0.803, and 0.785 in the Gravendeel dataset; 0.850, 0.885, 0.896, 0.865, and 0.864 in the TCGA dataset; 0.630, 0.735, 0.762, 0.764, and 0.779 in the Rembrandt dataset; and 0.847, 0.89, 0.897, 0.898, and 0.894 in the meta-cohort (Figures 4B, C; Supplementary Table S7). The performance of the GRMS in predicting prognosis was also evaluated in comparison with that of other clinical and molecular variables. As shown in Figures 4B, C and Supplementary Table S7, in terms of predictive accuracy, the GRMS outperformed other variables, including age, gender, WHO grade, IDH mutation, and chromosome 1p19q codeletion.

### Enrichment analysis

**FIGURE 5:**
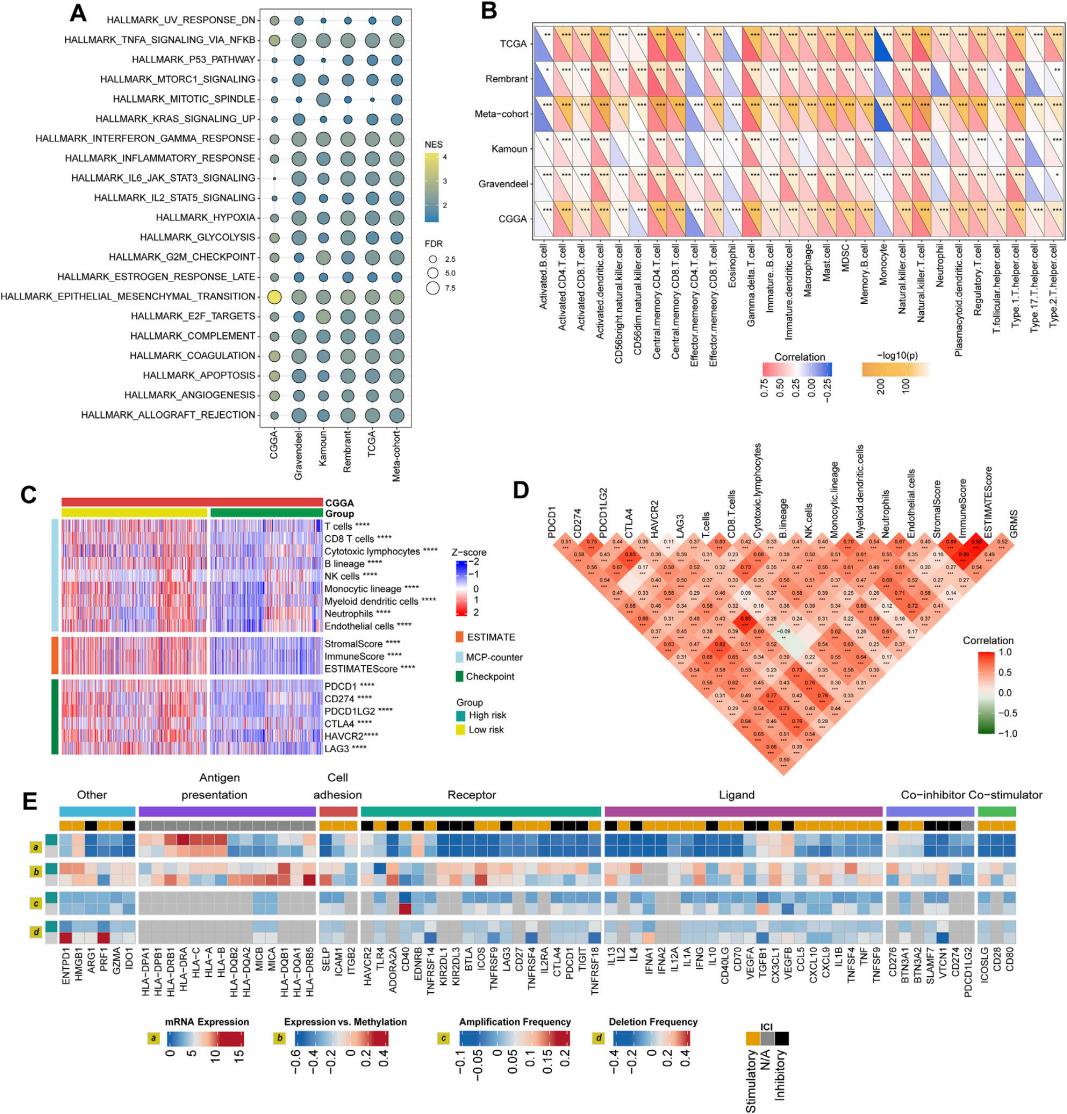
Immune landscape of the GRMS. **(A)** The dotplot visualizes the activation status of biological pathways between the two subgroups in the six datasets. Rows display the enriched terms, and columns represent the datasets. **(B)** Complex heatmap of correlation analysis of immune scores with GRMS groups. The red and blue colors indicate the positive and negative correlation coefficients, respectively. The yellow color represents the -log_10_-transformed *p*-value. The asterisks indicate the statistically significant *p*-values calculated by Spearman correlation analysis. **(C)** The heatmap demonstrates the immune infiltrates calculated using the MCP-counter, ESTIMATE methods, and expression differences of immune checkpoints between the two GRMS subgroups. All the data were z-score normalized from −2 to 2. **(D)** Correlations between the GRMS and immune infiltrates calculated using the MCP-counter, ESTIMATE methods and the expression levels of immune checkpoints. **(E)** Multi-omics analysis of the differences in immune checkpoints between the GRMS subgroups in the TCGA dataset. (*p < 0.05; **p < 0.01; ***p < 0.001; and ****p < 0.0001).

Through GSEA, we found that pro-tumor processes such as EMT, hypoxia, and the G2M checkpoint were relatively activated in the high-GRMS subgroup (Figure 4D; Supplementary Table S8). In addition, an increase in GRMS levels was likely to be responsible for the activation of the interferon-gamma (IFN-γ) response, interleukin 2 (IL2)/signal transducer and activator of transcription 5 (STAT5) signaling, and tumor necrosis factor a (TNFA) signaling via nuclear factor kappa B (NFκB). Furthermore, the top 20 dysregulated genes (FDR<0.05, |log_2_FC|>1) were displayed to validate the GSEA findings. We found that TIMP metallopeptidase inhibitor 1 (TIMP1), COL1A1, collagen type III alpha 1 chain (COL3A1), and insulin-like growth factor binding protein 2 (IGFBP2) were significantly upregulated in the EMT process. Cell division cycle 20 (CDC20) and ubiquitin-conjugating enzyme E2 C (UBE2C) were involved in the upregulation of the G2M checkpoint. The overexpression of vascular endothelial growth factor A (VEGFA) and serpin family E member 1 (SERPINE1) might contribute to the increase of TNFA signaling via NFκB and hypoxia in the tumor metabolism, and the upregulated expression of secreted phosphoprotein 1 (SPP1) and C-X-C motif chemokine ligand 10 (CXCL10) were related to the activation of the IL2/STAT5 response; the increase in the major histocompatibility complex, class II, DQ alpha 1 (HLA-DQA1), the major histocompatibility complex, class II, DM alpha (HLA-DMA), and C-C motif chemokine ligand 5 (CCL5) was associated with the upregulation of the IFN-γ response (Figure 4E). Finally, the GSEA on the remaining five sets also confirmed the results from the CGGA cohort (Figure 5A).

### Differences in GIME characteristics between GRMS subgroups

The purpose of the multi-omics analysis was to further explore the relationship between the immune response and the established GRMS. According to Danaher and colleagues’ immune infiltration data, we examined the distribution of immune cells between the high- and low-GRMS subgroups in detail. The high-GRMS group demonstrated a higher abundance of TILs, such as macrophages and CD8 T cells (*p* < 0.05; Supplementary Figure S7A), and demonstrated a strong correlation with the level of cytotoxic cells (cor = 0.54; *p* < 0.001), macrophages (cor = 0.58; *p* < 0.001), and exhausted CD8^+^ T cells (cor = 0.59; *p* < 0.001) (Supplementary Figure S7B). Immune signature scores were calculated using the ssGSEA method to validate the correlation between the GRMS and immune-infiltrating patterns. The boxplots revealed the high-immune infiltration group was significantly enriched in cases from the high-GRMS group (Supplementary Figures S8A–F). Furthermore, strong correlation levels between GRMS and glioma GIME contents were also observed, as displayed in Figure 5B, for example, gamma delta (γδ) T cell (TCGA, cor = 0.79; Rembrandt, cor = 0.74; meta-cohort, cor = 0.73; CGGA, cor = 0.71; Kamoun, cor = 0.65; and Gravendeel, cor = 0.56, *p* < 0.001, respectively), natural killer (NK) T cell (TCGA, cor = 0.79; Rembrandt, cor = 0.68; meta-cohort, cor = 0.67; CGGA, cor = 0.63; Kamoun, cor = 0.61; and Gravendeel, cor = 0.59, *p* < 0.001, respectively), and myeloid-derived suppressor cells (MDSCs) (TCGA, cor = 0.67; Rembrandt, cor = 0.55; meta-cohort, cor = 0.53; CGGA, cor = 0.53; Kamoun, cor = 0.52; and Gravendeel, cor = 0.39, *p* < 0.001, respectively). In addition, the GIME compositions from six independent glioma datasets with publicly available gene expression profiles were analyzed using the MCP-counter, a gene-expression-based TME deconvolution tool (Becht et al., 2016), and the ESTIMATE method. We compared the GIME distribution across different GRMS subgroups and found that the GIME composites differed significantly (Figure 5C). The levels of T cells, CD8^+^ T cells, cytotoxic lymphocytes, B lineage, NK cells, monocytic lineage, myeloid dendritic cells, neutrophils, and endothelial cells were significantly higher in the high-GRMS group and lower in the low-GRMS group (Figure 5C). A similar trend was obtained in the immune score calculated using the ESTIMATE algorithm. The expressions of immune checkpoint-related genes (Figure 5C), such as genes encoding programmed cell death 1 (PDCD1), programmed cell death 1 ligand 2 (PDCD1LG2), CTLA4, lymphocyte activating 3 (LAG3), and hepatitis A virus cellular receptor 2 (HAVCR2), were relatively higher in the high-GRMS group followed by the low-GRMS group. The aforementioned findings were consistent across the TCGA, meta-, Rembrandt, and Gravendeel cohorts, except the Kamoun dataset (Supplementary Figures S9A–E). In the Kamoun cohort, two checkpoints, namely, CD274 and HAVCR2, were upregulated in the high-GRMS group. Spearman correlation analysis revealed the consistent positive relation of the GRMS with monocytic linage infiltrates in the six enrolled sets (CGGA: cor = 0.37, *p* < 0.001; Gravendeel: cor = 0.30, *p* < 0.001; Rembrandt: cor = 0.22, *p* < 0.01; TCGA: cor = 0.33, *p* < 0.001; meta-cohort: cor = 0.35, *p* < 0.001; and Kamoun: cor = 0.24, *p* < 0.01). Moreover, except the Rembrandt set, as to the immune checkpoints, the estimated GRMS demonstrated a strong correlation with CD274 expression level in the CGGA set (cor = 0.39, *p* < 0.001), Gravendeel set (cor = 0.45, *p* < 0.001), TCGA set (cor = 0.54, *p* < 0.001), meta-cohort (cor = 0.41, *p* < 0.001), and Kamoun (cor = 0.35, *p* < 0.01), which indicated that patients in the high-GRMS group might respond to anti-tumor immunotherapy (Supplementary Figures S10A–E).

### Regulation of immunomodulators

We examined immunomodulator (IM) gene expression, somatic copy number alterations (SCNAs), and expression control via epigenetic and miRNA mechanisms. The gene expression of IMs (Figure 5D) varied across risk subtypes, and IM expression largely segregated tumors by immune subtypes, perhaps indicative of their role in shaping the TME. Genes with significant differences between subtypes, including CXCL10, CD80, CCL5, and the HLA family, were highly expressed in the high-risk subgroup (*p* < 0.001). DNA methylation of many IM genes, e.g., toll-like receptor 4 (TLR4), IL10, PDCD1LG2, endothelin receptor type B (EDNRB), TNFRSF18, and integrin subunit beta 2 (ITGB2), was observed in the high-GRMS group, while IL10, ITGB2, CD28, PDCD1, HLA-B, CD276, VEGFA, PDCD1LG2, EDNRB, perforin 1 (PRF1), CD274, and CD40 inversely correlated with gene expression, suggesting epigenetic silencing (Figure 5D).

### Potential of the GRMS as an effective indicator for immunotherapy

**FIGURE 6:**
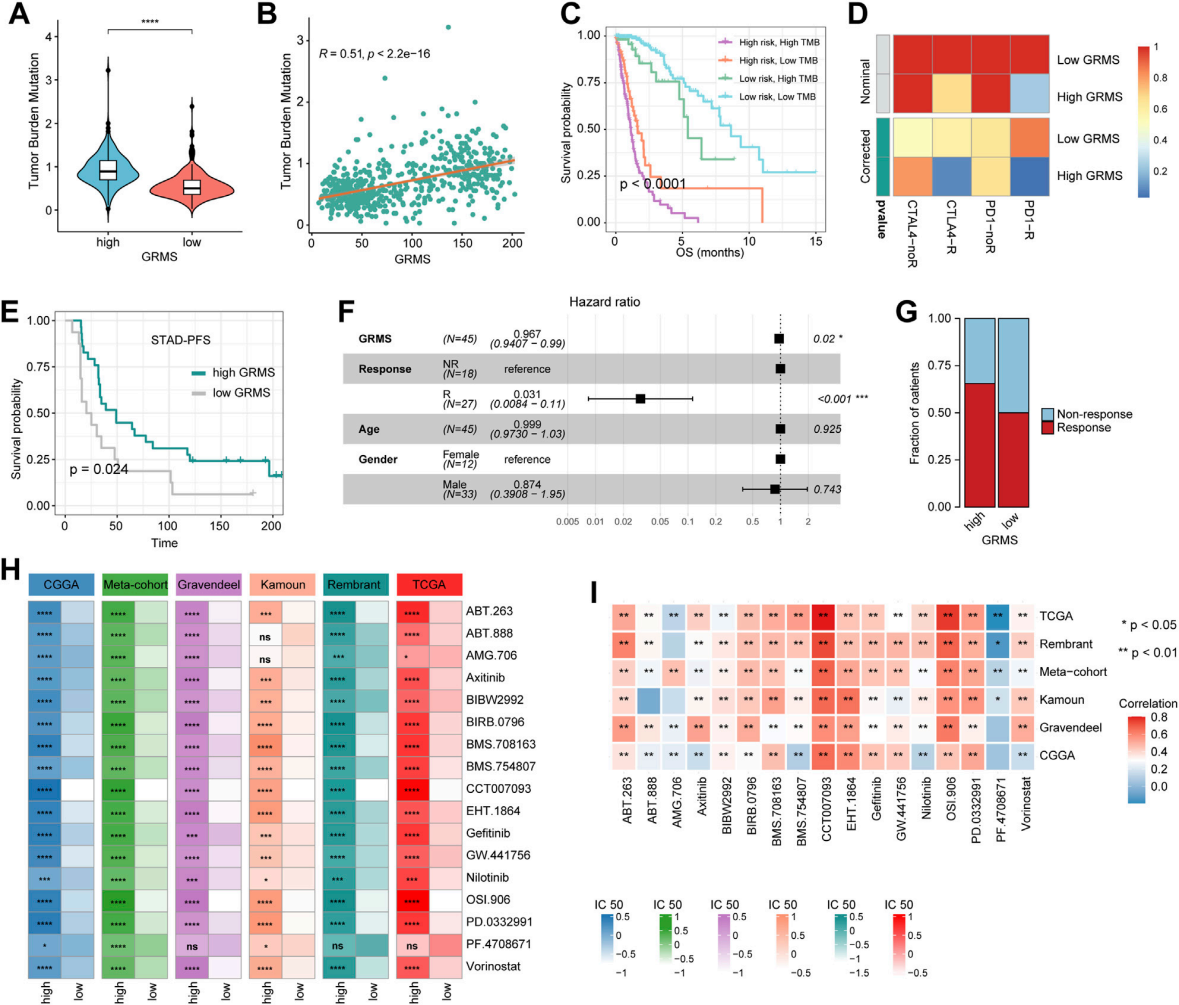
Predictive value of the GRMS in immunotherapy response. **(A)** The violin plot demonstrates that there were significant TMB differences after being grouped by GRMS values in gliomas. **(B)** The dotplot demonstrates the close correlation of GRMS values with TMB values. **(C)** The K–M plot of gliomas; OS stratified by TMB and GRMS values. **(D)** The submap algorithm predicts the probability of anti-PD1 and anti-CTLA4 immunotherapy response in high- and low-GRMS groups. **(E)** The K–M plot demonstrates a significant difference in the survival rate between a high or low GRMS in the immunotherapy-applied set. **(F)** Multivariate Cox analysis reveals that the GRMS signature was an independent predictor for STAD patients after receiving immunotherapy. **(G)** The stacked histogram displays the difference in anti-tumor immunotherapeutic responsiveness between a high and low GRMS. **(H)** Heatmaps demonstrate the differences in IC_50_ values of drugs between the high- and low-GRMS groups. **(I)** Correlation of IC_50_ values of drugs with GRMS values in the six datasets. Rows display the datasets, and columns represent the different drugs. The red and blue colors indicate the positive and negative correlation coefficients, respectively (*p < 0.05; **p < 0.01; ***p < 0.001; and ****p < 0.0001).

Since the development of the GRMS was immune-related, we assumed that there were differences in immune characteristics and immunotherapeutic effects at different levels of the GRMS. Tumor mutation burden (TMB) plays a crucial role in clinical practice, and an intrinsic relationship was detected between TMB and GRMS. TMB values were markedly increased in patients with a higher GRMS, as shown in Figure 6A (*p* < 0.001). Using Spearman correlation analysis, the GRMS was positively correlated with the TMB value of gliomas (cor = 0.51, *p* < 0.001, Figure 6B), indicating gliomas with a relatively high GRMS may be responsive to anti-tumor immunotherapy. Given the strong association between TMB and the GRMS, we calculated the synergistic impact of both variates on glioma outcomes. The stratification analysis demonstrated that even when TMB values interfered, the prognostic signature remained an independent predictor for glioma prognosis (log-rank test, *p* < 0.001, Figure 6C). Therefore, these findings revealed that the GRMS might serve as a potential prognosis predictor independent of TMB and as an effective tool to screen patients who will benefit from immunotherapy. The submap algorithm was used to predict the response of high- and low-GRMS groups to anti-PD1 and anti-CTLA4 immunotherapy. We found that the high-GRMS group might derive greater benefits from anti-PD1 therapy (Bonferroni corrected *p* = 0.013, Figure 6D). For further investigation into the association of the GRMS with immunotherapy, OS and progression-free survival (PFS) were compared between the two risk subgroups. We found that the gliomas with higher GRMS values demonstrated better outcomes in PFS and OS (Figure 6E, and Supplementary Figure S11A). In addition, the GRMS could predict the PFS, independent of age, gender, and immunotherapeutic response, calculated using multivariate Cox regression analysis (Figure 6F). However, consistent findings of multivariate Cox analysis were not found in predicting OS of gliomas (Supplementary Figure S11B). As shown in Figure 6G, patients with a higher GRMS value were more likely to benefit from immune checkpoint treatment.

### GRMS predicts the response to anti-tumor adjuvant therapy

A correlation analysis was conducted between glycosyltransferase-related mRNAs and drug sensitivity. The data showed that most drugs had a synergistic effect with genes, such as PYGL, HAS2, GALNT10, EXT2, EXT1, B4GALT5, B4GALT4, B4GALT1, and B3GNT9, while ST8SIA3, PYGM, MFNG, GLT1D1, and ALG5 might have a strong antagonistic effect on the drugs presented in Supplementary Figure S11C. In addition, the R package “Ridge” was introduced to assess the IC_50_ value of drugs on the expression data of RNA-seq samples. The drugs that were common among the six sets with statistical significance were selected for further analysis. The IC_50_ values indicated that PD-0332991 (which targets cyclin-dependent kinase 4/6, CDK4/6), axitinib (an inhibitor of VEGFR), BIBW2992 (an irreversible inhibitor of the ErbB family of tyrosine kinases), BIRB0796 (an inhibitor of mitogen-activated protein kinase, MAPK), and gefitinib (an inhibitor of epidermal growth factor receptor, EGFR) might be alternatives in treating gliomas in the high-risk group (Figure 6H). The Spearman analysis further validated the positive correlation of GRMS values with IC_50_ values of PD-0332991, axitinib, BIBW2992, BIRB0796, and gefitinib (*p* < 0.05, respectively, Figure 6I). Adjuvant radiotherapy (ART) and adjuvant chemotherapy (ACT) are currently the preferred treatments for gliomas and have significant anti-glioma activity (Rao et al., 2017). Therefore, we assessed whether the application of adjuvant therapy could affect the ability of the GRMS to predict the clinical outcomes of gliomas. The survival advantage was observed in patients treated with ART or ACT who had low GRMS (*p* < 0.001, Supplementary Figures S11D, E).

### Correlation between GIME features and the cancer somatic genome

**FIGURE 7:**
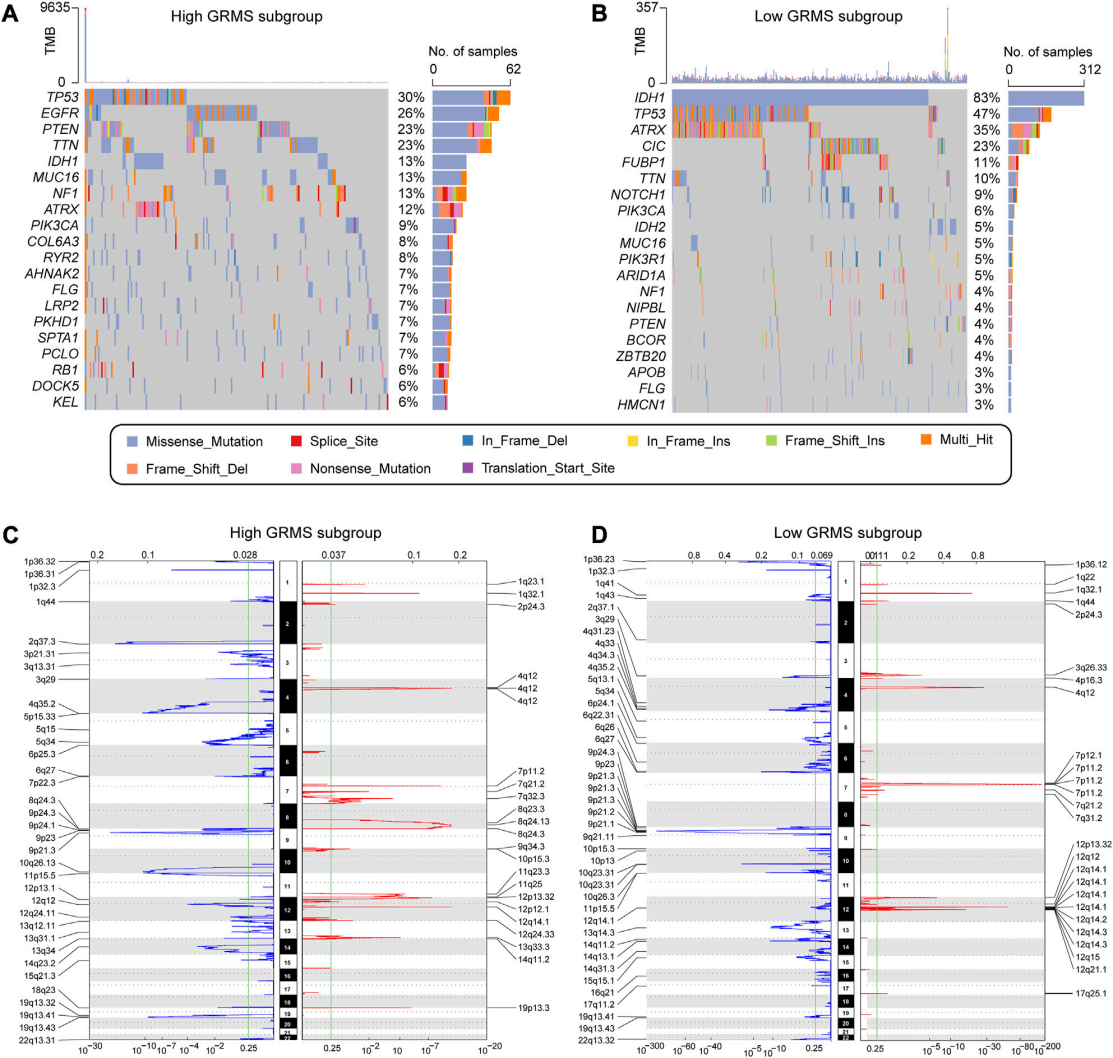
Correlation between the GRMS and genomic variants. The waterfall plots of gene mutation frequency in high- **(A)** and low- **(B)** GRMS groups. Composite copy number profiles in gliomas with gains in red, losses in blue, and gray highlighting differences in high- **(C)** and low- **(D)** GRMS groups.

The somatic variations in the TCGA glioma cohort between GRMS subgroups were analyzed using the maftools package. The top 20 altered genes are shown in Figures 7A, B. In the high-GRMS group, tumor protein P53 (TP53) demonstrated the highest mutation percentage (30%). In the low-GRMS group, IDH1 showed the highest mutational frequency (83%). By GISTIC 2.0 analysis, we found that there were significant aberrations in total CNV levels for patients in the two groups. For patients in the high-GRMS group, copy number deletion was found mainly on 1p, 2q, 6q, 9p, and 10q. Copy number gain was observed predominantly on chromosomes 7p, 12q, and 19p (Figure 7C). Among patients in the high-GRMS group, copy number deletion was found to be mainly located at 1p, 4q, 9p, and 10q. Copy number amplification was found to be mainly located at 7p and 12q (Figure 7D). In addition, compared with the low-risk group, focal amplification peaks were observed for well-characterized cancer-related genes HAS2 (8q24.13) and PDGFRA (4q12), which might be the reason for the poor outcomes in the high-risk group. In summary, the CNV levels in the two subgroups were significantly distinct.

## Discussion

Among the cancers of the central nervous system, GBMs have the most aggressive subtypes, characterized by poor outcomes and ineffective treatments (Gusyatiner and Hegi, 2018). To overcome the significant heterogeneity in gliomas, molecular profiles have been used to identify the homogeneous subtypes (Nicholson and Fine, 2021). The importance of glycosyltransferase in tumor immunity has become increasingly clear, but the mechanisms by which glycosyltransferase molecules influence glioma prognosis remain obscure (Rudd et al., 2001; Li X. et al., 2020).

Here, we analyzed the genetic and expressive characteristics of 40 glycosyltransferase-related DEGs and confirmed that the imbalance in marker expression might be associated with the dysregulation of genome variation. Our research established a comprehensive computational method to identify a prognostic GRMS with significant stability and robustness through an integrated analysis of 2,030 glioma cases from publicly available datasets. First, glycosyltransferase-associated molecules, which were differentially expressed between glioma and normal brain tissues, were screened out. Second, 80 combinations of 10 machine learning methods were introduced, and the combination of Lasso and random forest was identified as the optimal one with the maximum c-index value, which remarkably reduced the dimensionality of variables and contributed to an accurate mathematical statistical model. Among the 18 most valuable mRNAs identified for the GRMS, one mRNA has been reported to be related to gliomas. The overexpression of PYGL in GBM tissues was associated with poor survival. Knockdown of PYGL decreased the growth and survival of the GBM cell line (Zois et al., 2022). Our comprehensive analyses revealed that the GRMS was a hazardous biomarker for the prognosis of glioma patients, consistent with previous findings. The calculated AUC values identified that the GRMS could accurately predict 1-, 2-, 3-, 4-, and 5-year clinical outcomes of gliomas. Based on the stability assessment of the GRMS across the six datasets, it could be concluded that the GRMS has significant potential as an independent biomarker for clinical applications.

By promoting the metabolism of the glioma cells, metabolic plasticity not only promoted energy generation and substrate synthesis but also induced immune evasion (Ganapathy-Kanniappan, 2017; Venneti and Thompson, 2017). For example, GIME remodeling, including glioma-associated microglia/macrophages (GAMMs), T cells, NK cells, and myeloid-derived suppressor cells (MDSCs), could lead to glioma progression and development instead of surveillance and improving the prognosis (Qiu et al., 2021). It has been established that reprogramming the GIME could influence the treatment of brain tumors and increase the effectiveness of immunotherapy (Gieryng et al., 2017). In the present research, there was a positive correlation between the GRMS and the infiltration level of immune cells, and significant differences in clinical characteristics and infiltration levels of macrophages and CD8^+^ T cells were observed between different GRMS groups. In addition, the GRMS was reliable in comprehensively assessing immune-associated molecules. For example, the GRMS displayed a positive association with the expression of immune checkpoints, such as HAVCR2 and CD274. As a ligand for the inhibitory receptor PDCD1, CD274 could mediate anti-tumor immune escape by regulating the activation threshold of T cells and limiting T-cell effector responses (Jiang et al., 2019). Furthermore, it has been demonstrated that HAVCR2 is an inhibitory receptor expressed on innate immunity cells, such as macrophages and dendritic cells, as well as on the T cells producing IFN-g and FoxP3+ Treg cells. It suppresses the immune response when the ligand interacts with HAVCR2 (Das et al., 2017). Therefore, we hypothesized that the GRMS might be related to GIME reprograming. It is also recognized that the crucial feature of an inflamed GIME is the upregulation of immunoinhibitory checkpoints (Wu et al., 2019). Here, we confirmed that the immune checkpoints were remarkably overexpressed in the high-GRMS subgroup, which might be due to the elevated infiltrates of immune cells, demonstrating that gliomas with high GRMS might have increased sensitivity to anti-tumor immunotherapy. Recent research reported that the inhibition of immune checkpoints could contribute to the suppression of tumor growth by restoring the cytotoxicity of T lymphocytes (Barry and Bleackley, 2002; Núñez Abad et al., 2022). Immunotherapeutic strategies with monoclonal antibodies have been confirmed to be clinically proven to be effective, with significant responses and acceptable side effects in several types of tumors, such as muscle-invasive bladder cancer (MIBC), melanoma, and non-small-cell lung cancer (NSCLC) (Schmid-Bindert and Jiang, 2015; Tsai and Daud, 2015; Bellmunt et al., 2017). However, the sensitivity of gliomas to immunotherapy differs remarkably, with a few subjects obtaining complete remission (CR) and others demonstrating no or low clinical benefits (Zhao et al., 2022). In the present research, the GRMS was validated to be significantly associated with the response of gliomas to immune checkpoint inhibitor (ICI) therapy through the submap algorithm; that is, the GRMS could stratify ICI-treated patients into responder and non-responder subgroups. A high-GRMS value implied elevated response and sensitivity to ICI therapy, which demonstrated that the clinical application of the GRMS might contribute to the process of clinical decision making for the treatment of gliomas. Research has shown that there is a connection between different glycosyltransferase subtypes and variations in mRNA transcriptomes, specifically related to immune-related biological pathways and drug responses. In the present research, TNFA via the NFκB signaling pathway, the IFN-γ response, hypoxia, the G2M checkpoint, and the EMT process using the GSEA were positively correlated with the GRMS. TNF α, also referred to as tumor necrosis factor, is a cytokine that possesses the ability to directly eliminate tumor cells, elevated expression levels of inflammatory signatures, and innate immunity (Pegoretti et al., 2018). Recent research suggests that selective reduction of the TNF cytotoxicity threshold could increase the susceptibility of tumors to immunotherapy, which might contribute to the sensitivity to immunotherapeutic interventions for patients with high GRMS (Vredevoogd et al., 2019). IFN-γ is a cytokine predominantly secreted by various immune cells, encompassing NK cells and CD8 cytotoxic T lymphocytes (CTLs). The upregulated IFN-γ response could activate the JAK–STAT signaling pathway and promote the expression of chemokines and antigen-presenting molecules (including MHC molecules) (Ivashkiv, 2018). In addition, the increased IFN-γ response could also stimulate a type I immune response characterized by M1 macrophage polarization (Glass and Natoli, 2016). Recent reports demonstrate that M1 phenotypic macrophage activation is characterized by the upregulation of inducible nitric oxide synthase (iNOS) and interleukins IL6 or IL12 and the enhancement of the Th1 immune response (Mills et al., 2000; Stout et al., 2005). Our findings demonstrated that the samples with high-GRMS values exhibited activation of the IFN-γ response, which might be responsible for the samples with high-GRMS values being sensitive to immunotherapy. The upregulated and/or promoted processes of hypoxia, the G2M checkpoint, the EMT process, and the features of tumors in the high-GRMS subgroup might be the reasons for the disadvantageous outcomes of gliomas (Liu et al., 2019; Liu et al., 2020).

The positive correlation between glycosyltransferase and tumor immunity has been recognized (Dusoswa et al., 2020; Jalali Motlagh et al., 2021). Glycosyltransferase alterations could change the nature of the TME and reverse adverse characteristics of TME cells, altering a “cold tumor” into a “hot tumor.” For example, the changed glycosyltransferase motifs on Mucin 1 (MUC1) could promote cancer immune surveillance by interacting with CD169, which enhances macrophage activation after binding to sialylated MUC1 and promotes tumor growth (Reily et al., 2019). Moreover, a decrease in the levels of UDP-GlcNAc and consequently in the levels of complex branched N-glycans promotes proinflammatory T helper 17 over anti-inflammatory-induced regulatory T-cell differentiation (Araujo et al., 2017). Therefore, it is imperative to classify tumors according to their glycosyltransferase phenotypes, especially considering glycosyltransferase’s role in the immune regulation of gliomas.

The effectiveness of immunotherapeutic treatment relies on the infiltrates of CD8^+^ T cells in tumor nests, and ICIs are more effective when combined with therapy that increases the number of CD8^+^ T cells (Yang et al., 2021). In this research, using the ssGSEA and ESTIMATE algorithms, we found that the CD8^+^ T-cell infiltration level was positively correlated with the GRMS value. Samples in the high-GRMS group demonstrated higher levels of infiltrating CD8^+^ T cells according to deconvolution methods and exhibited higher levels of markers related to co-stimulators, HLA, interferons, and chemokines. Our study also found a remarkable correlation between the GRMS and immune checkpoint expression levels, and validated samples with higher GRMS had higher checkpoint molecule expression. It has been recognized that patients with an overexpression of the PD-L1 checkpoint generally demonstrate significant sensitivity to immunotherapy (Sánchez-Magraner et al., 2020). Therefore, we hypothesized that the combination of ICIs and glycosyltransferase modifiers might have tremendous potential in contributing to the development of new therapeutic strategy regimes. Previous studies have confirmed that glycosyltransferase-related genes play an important role in chemotherapy (Mereiter et al., 2019). Based on the drug sensitivity analysis by the GDSC database and CGGA set, it might be validated that the GRMS could help guide optimal chemotherapy treatment. In clinical practice, the GRMS might be used to assess the expressive patterns of glycosyltransferase-related makers and the corresponding infiltrate features of GIME in gliomas to predict the outcomes of objects and guide the clinical decision-making process.

It is noteworthy that the GRMS has clinical significance in gliomas, but there are still some limitations. First, all datasets enrolled in the present research were obtained from retrospective studies conducted in a single center. In order to validate the GRMS, multicenter prospective research should be carried out. Second, further investigation is needed to determine the underlying mechanisms through which valuable glycosyltransferase-related mRNAs impact the GIME and immunotherapeutic responsiveness. Finally, more immunotherapy cohorts are urgently required to validate the response to anti-tumor immunotherapy in gliomas.

## Conclusion

We comprehensively analyzed glycosyltransferase-related mRNAs in gliomas and established a prognostic marker named the GRMS. The calculated GRMS might be a suitable candidate signature for assessing clinical prognosis and has significant potential to accelerate the development of antitumor treatment strategies for gliomas.

## Data Availability

The original contributions presented in the study are included in the article/Supplementary Material; further inquiries can be directed to the corresponding author.
