# Supplementary figures and images for: Machine learning-based identification of glycosyltransferase-related mRNAs for improving outcomes and the anti-tumor therapeutic response of gliomas
Source: Front Pharmacol. 2023 Aug 16;14:1200795. doi: 10.3389/fphar.2023.1200795 (PMC10468601; doi:10.3389/fphar.2023.1200795)

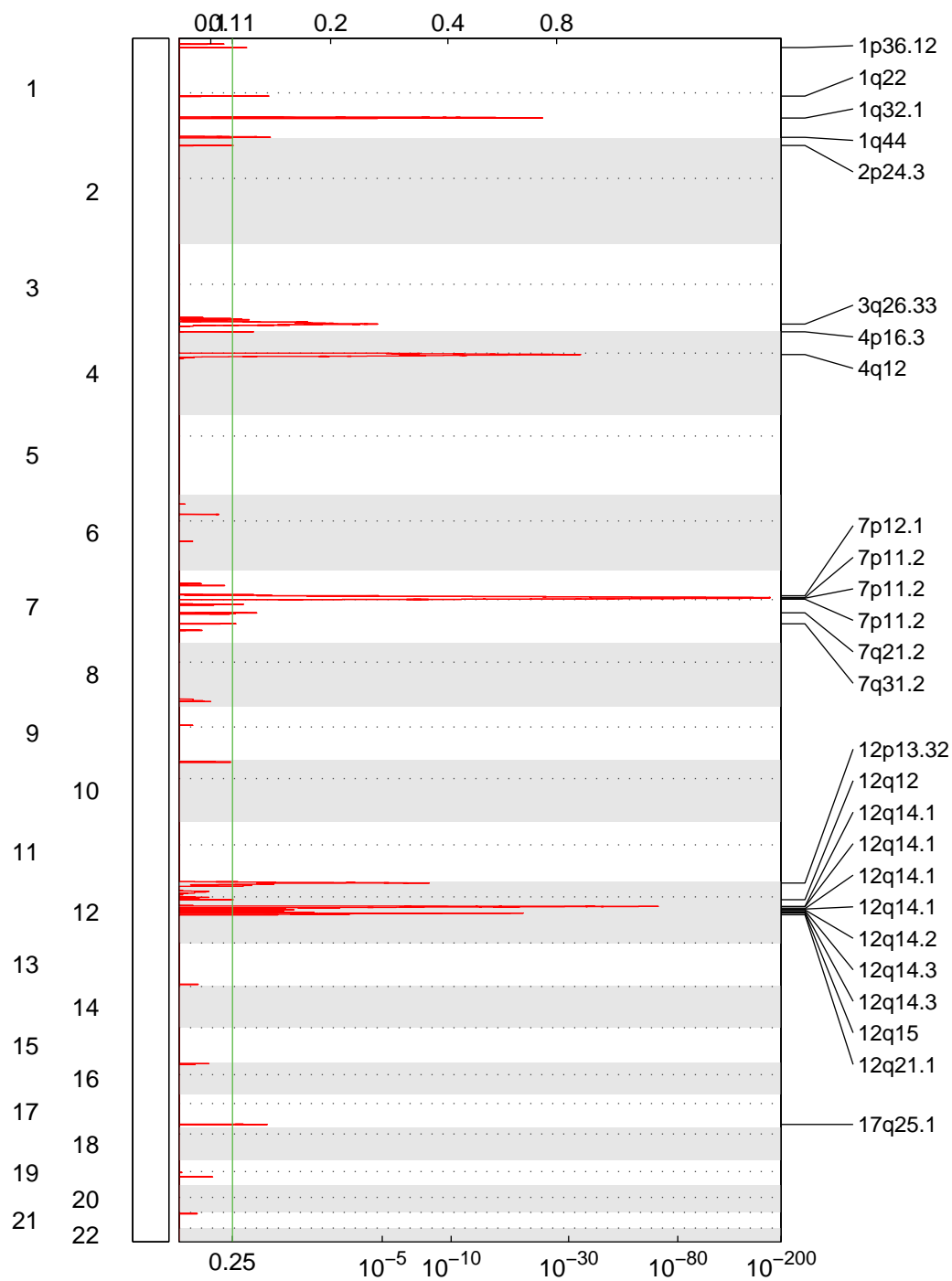

Supplement: Supplementary file 1 [file DataSheet3.zip › cnv/490127/amp_qplot.pdf]

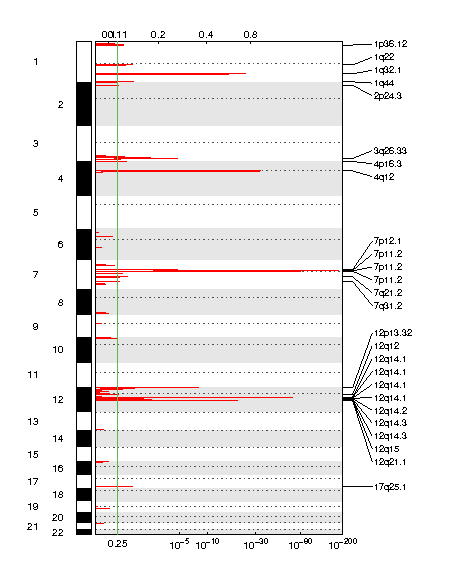

Supplement: Supplementary file 1 [file DataSheet3.zip › cnv/490127/amp_qplot.png]

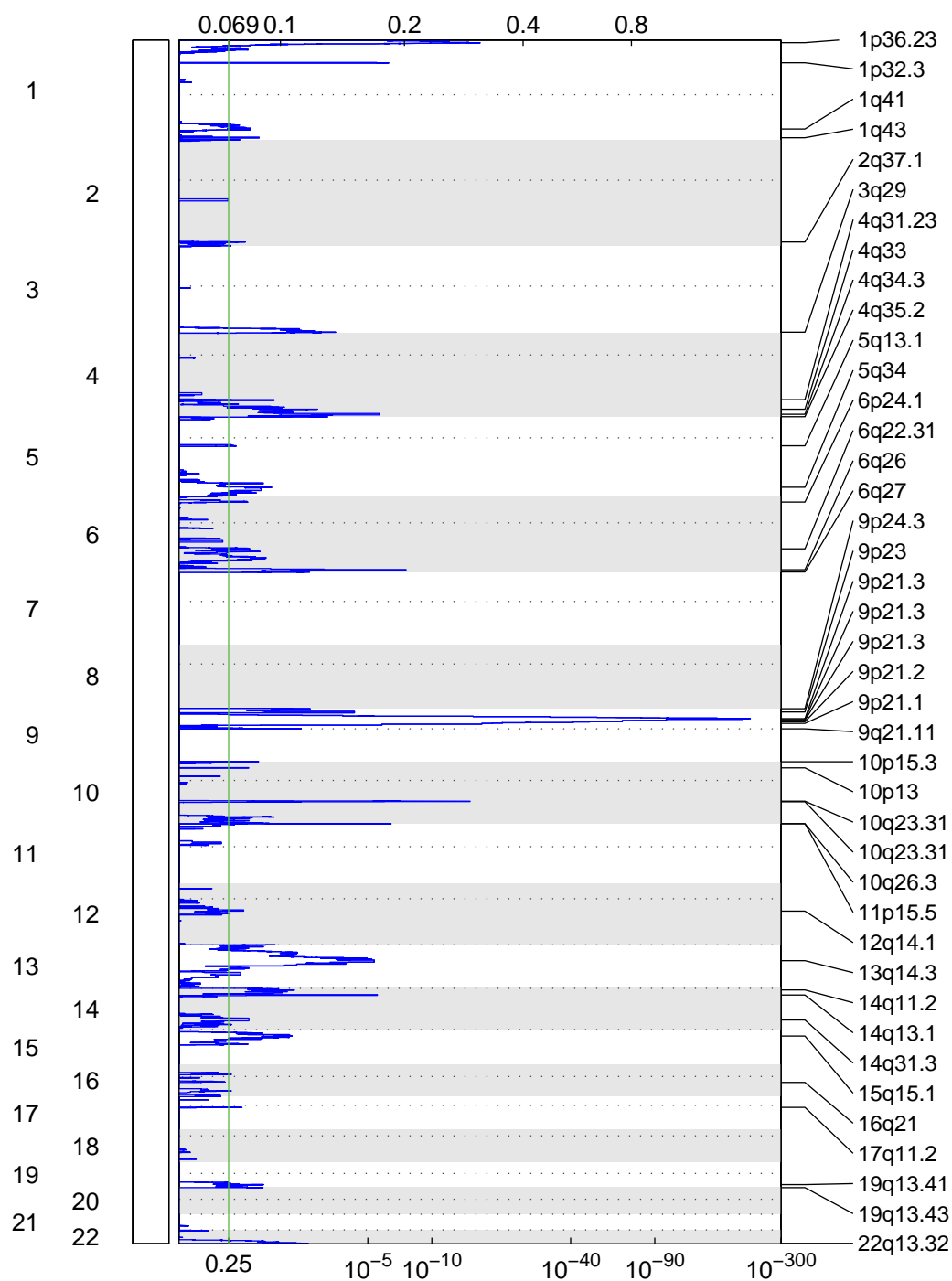

Supplement: Supplementary file 1 [file DataSheet3.zip › cnv/490127/del_qplot.pdf]

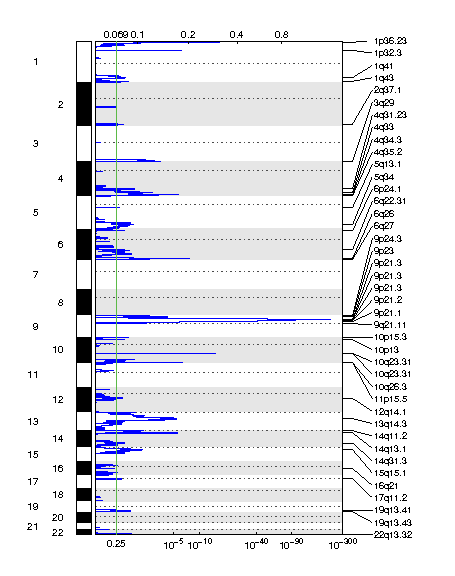

Supplement: Supplementary file 1 [file DataSheet3.zip › cnv/490127/del_qplot.png]

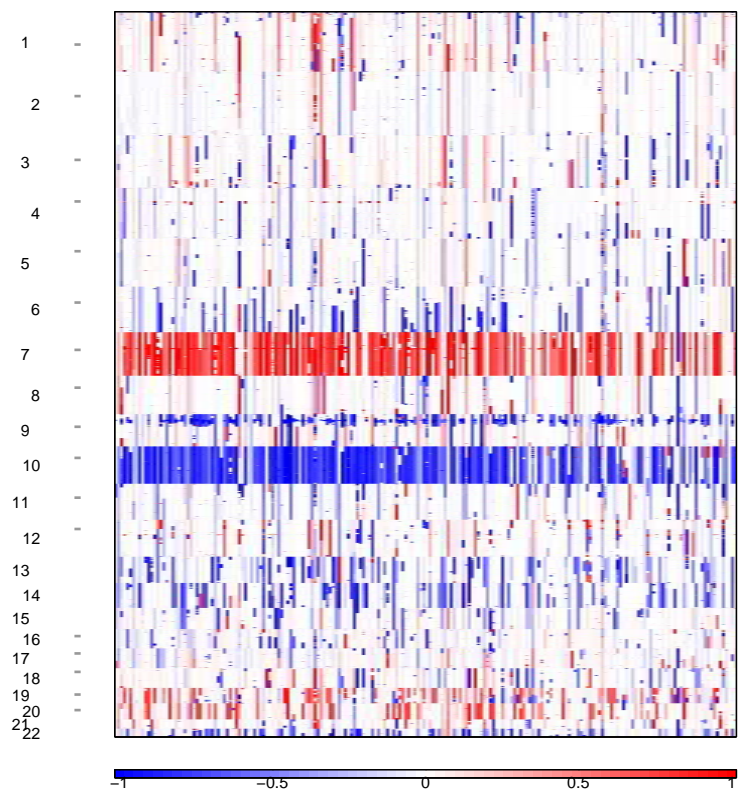

Supplement: Supplementary file 1 [file DataSheet3.zip › cnv/490127/raw_copy_number.pdf]

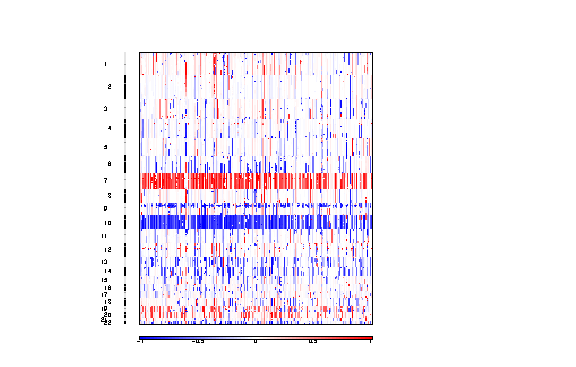

Supplement: Supplementary file 1 [file DataSheet3.zip › cnv/490127/raw_copy_number.png]

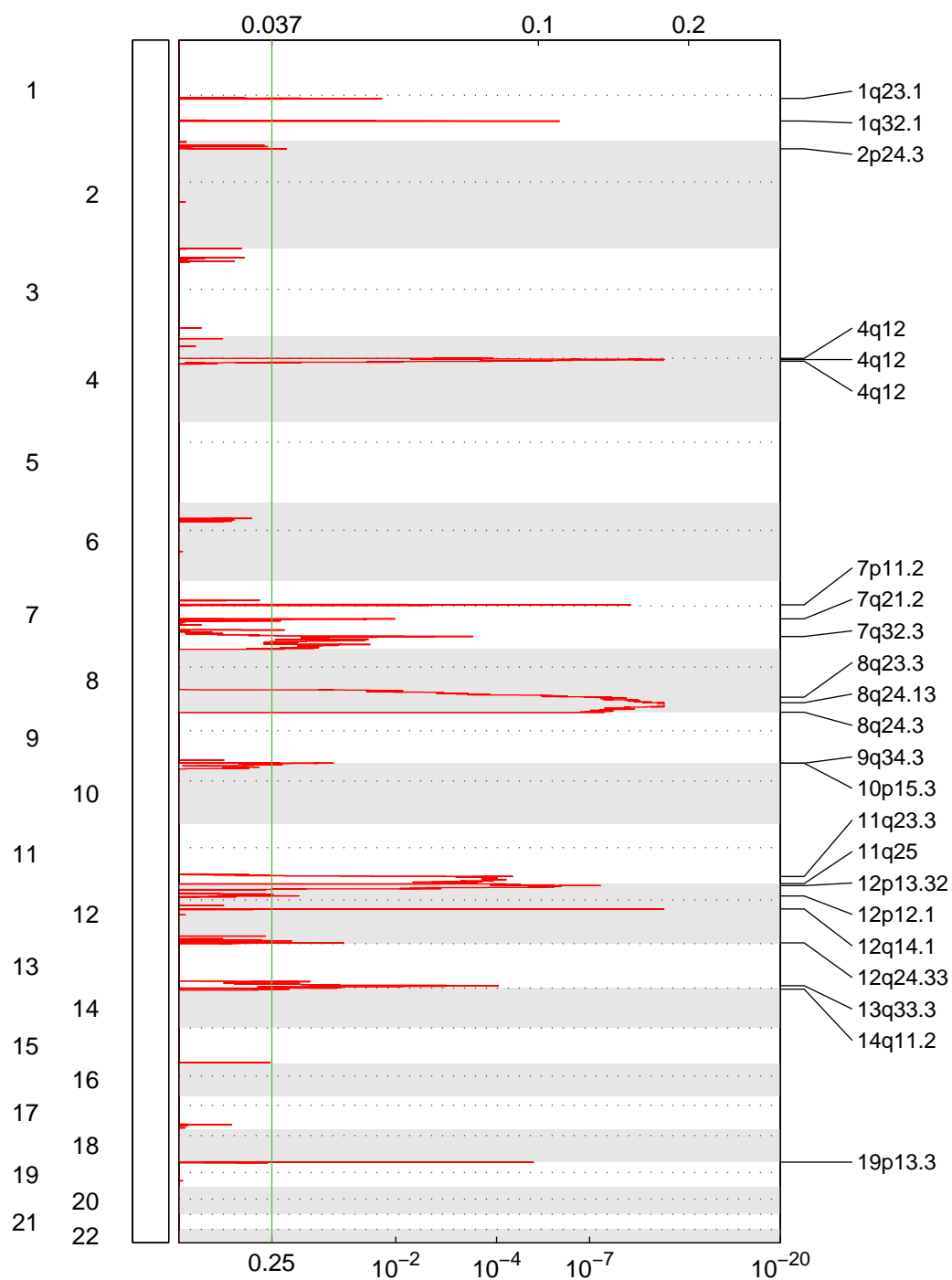

Supplement: Supplementary file 1 [file DataSheet3.zip › cnv/490406/amp_qplot.pdf]

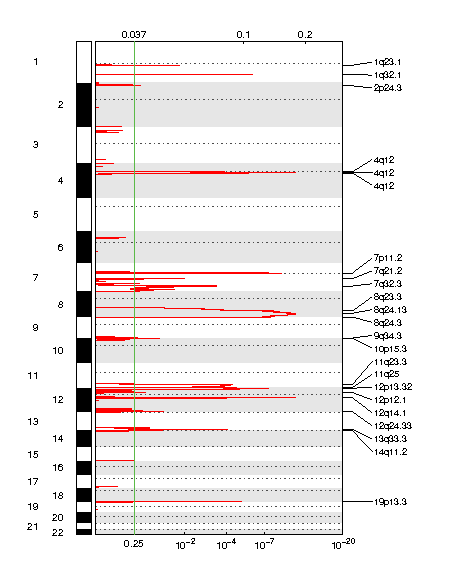

Supplement: Supplementary file 1 [file DataSheet3.zip › cnv/490406/amp_qplot.png]

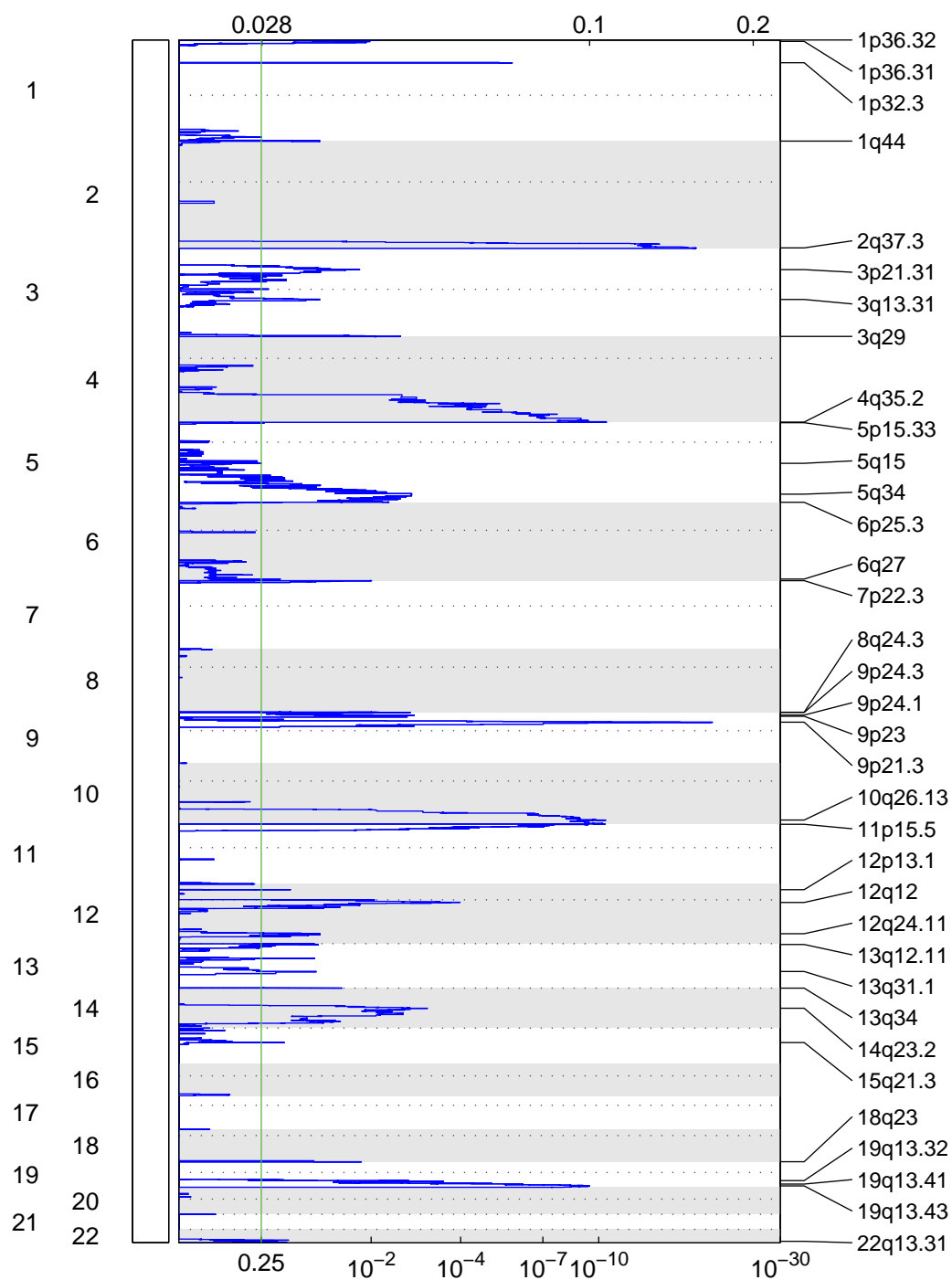

Supplement: Supplementary file 1 [file DataSheet3.zip › cnv/490406/del_qplot.pdf]

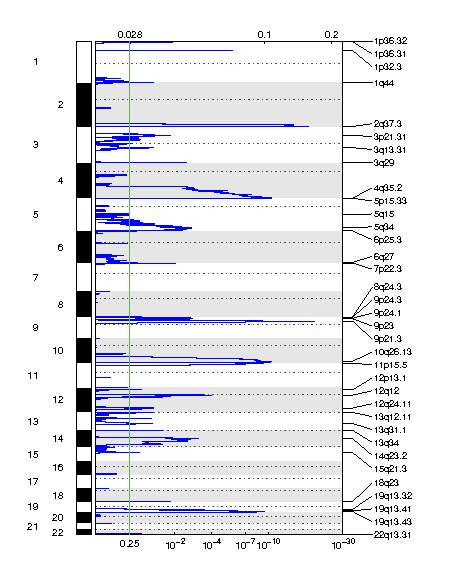

Supplement: Supplementary file 1 [file DataSheet3.zip › cnv/490406/del_qplot.png]

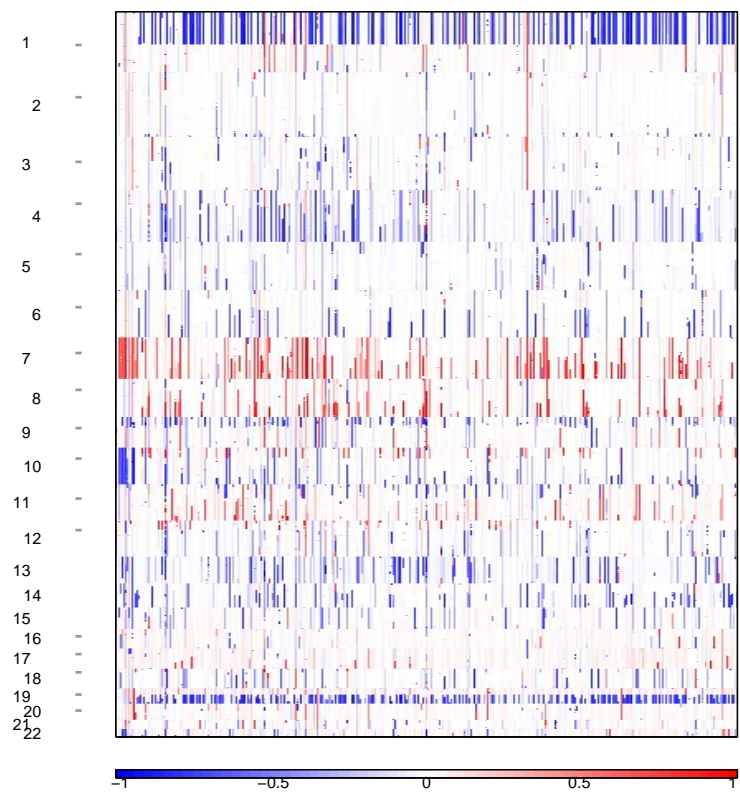

Supplement: Supplementary file 1 [file DataSheet3.zip › cnv/490406/raw_copy_number.pdf]

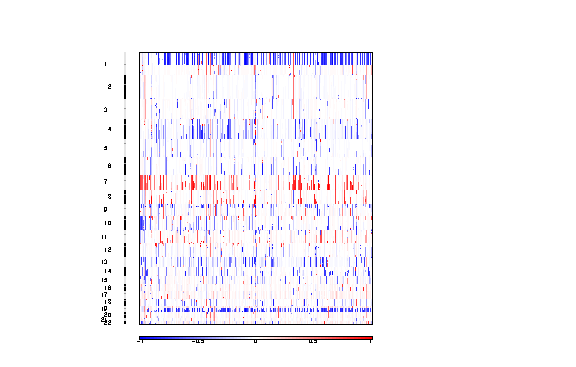

Supplement: Supplementary file 1 [file DataSheet3.zip › cnv/490406/raw_copy_number.png]
