# Supplementary material for: Machine learning-based identification of glycosyltransferase-related mRNAs for improving outcomes and the anti-tumor therapeutic response of gliomas
Source: Front Pharmacol. 2023 Aug 16;14:1200795. doi: 10.3389/fphar.2023.1200795 (PMC10468601; doi:10.3389/fphar.2023.1200795)
Supplement: Supplementary file 2 [file DataSheet1.docx]

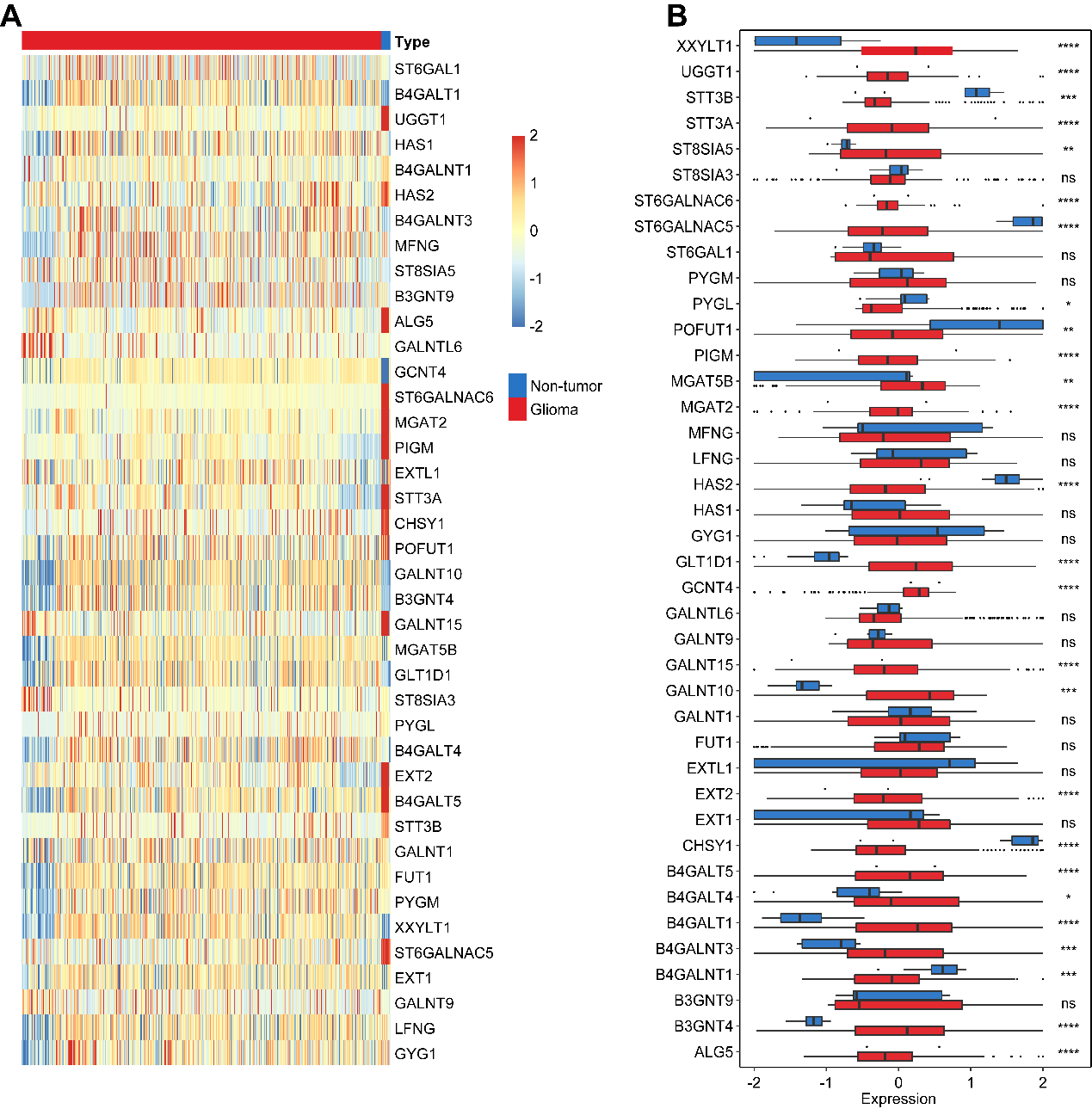


**Figure S1**. Heatmap of distribution of glycosylation-related molecules (A). Barplot demonstrated there were 25 genes differentially methylated between gliomas and non-tumor samples (B).


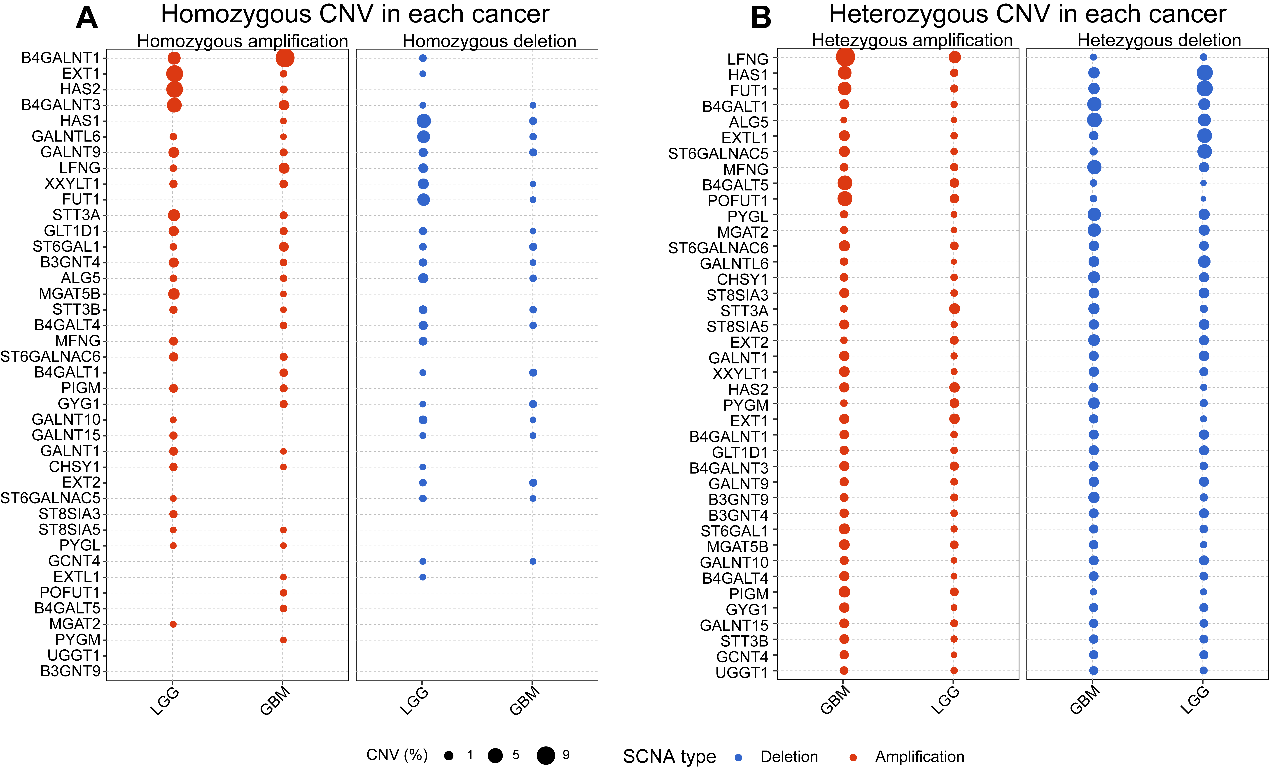


**Figure S2**. The CNV bubble chart shows the distribution of (A) homozygous and (B) heterozygous CNV of glycosylation-related molecules in brain cancer. Hete Del: Heterozygous deletion; Homogeneous amplification: Homozygous amplification; Homo Del: Homozygous deletion.


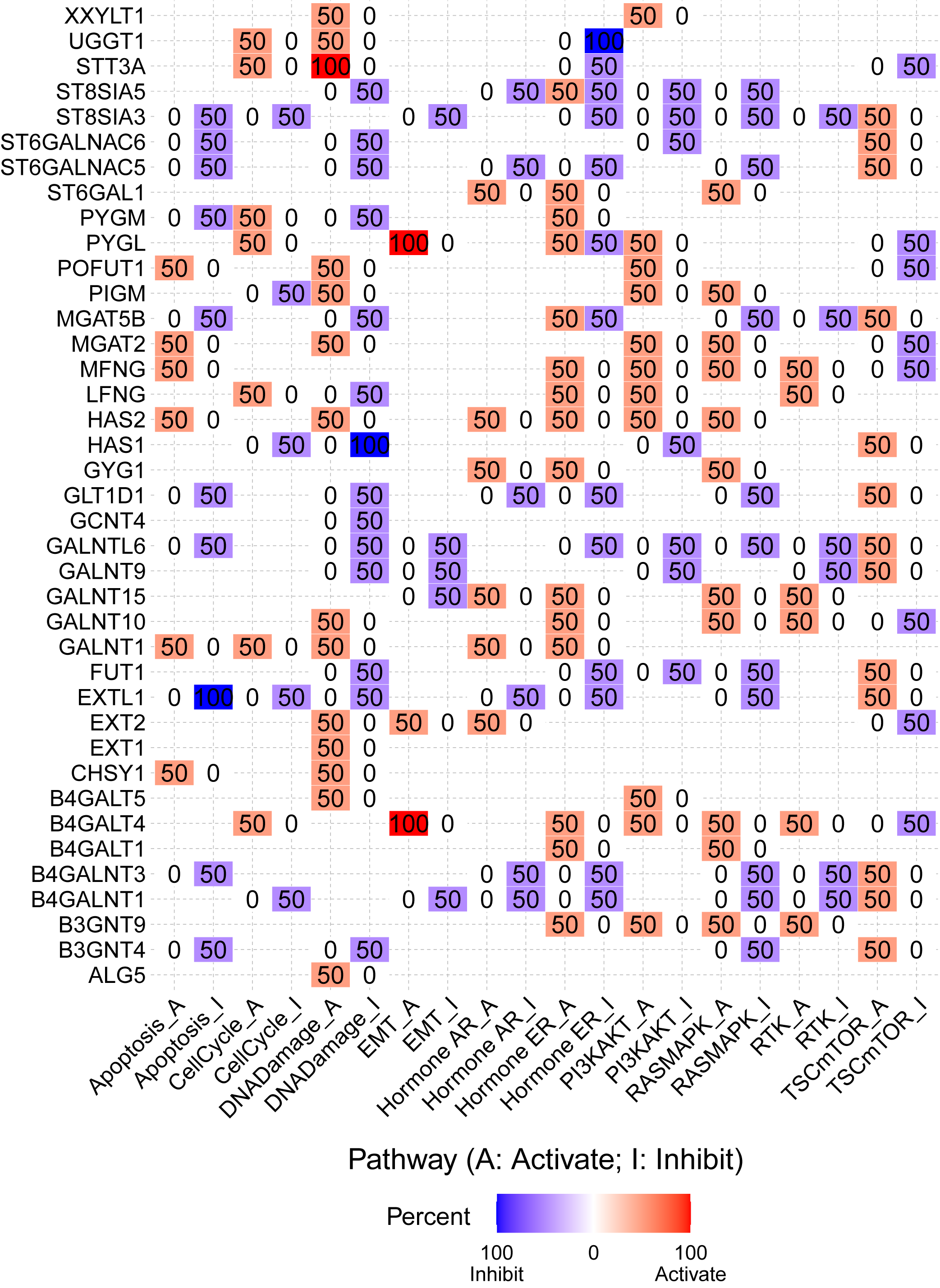


**Figure S3.** Heatmap of correlation analysis of genes with 10 cancer-related pathways in protein level based on the online tool-GSCA.


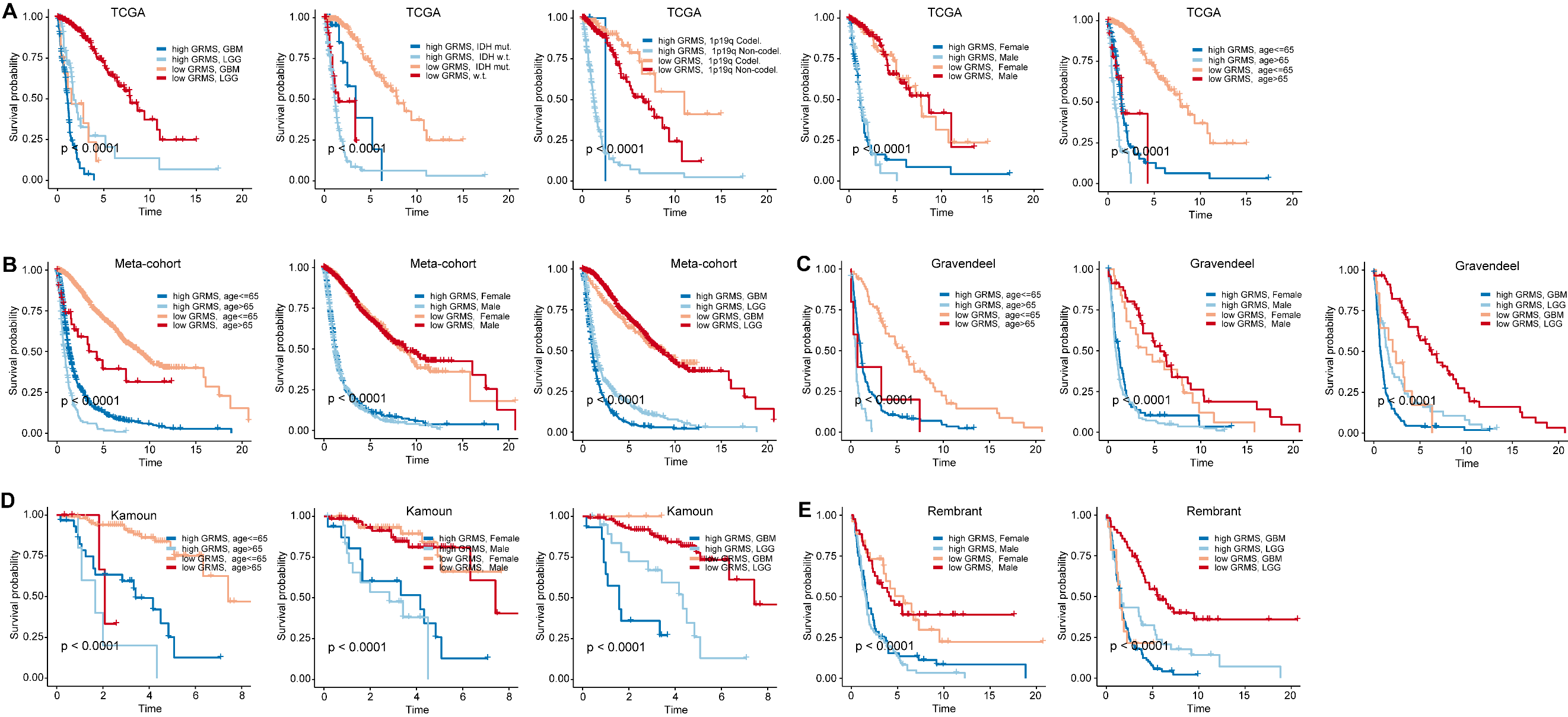


**Figure S4.** Kaplan-Meier curves demonstrated significant advantages in low GRMS subgroup for patients of different age, gender, IDH, and 1p19q status.


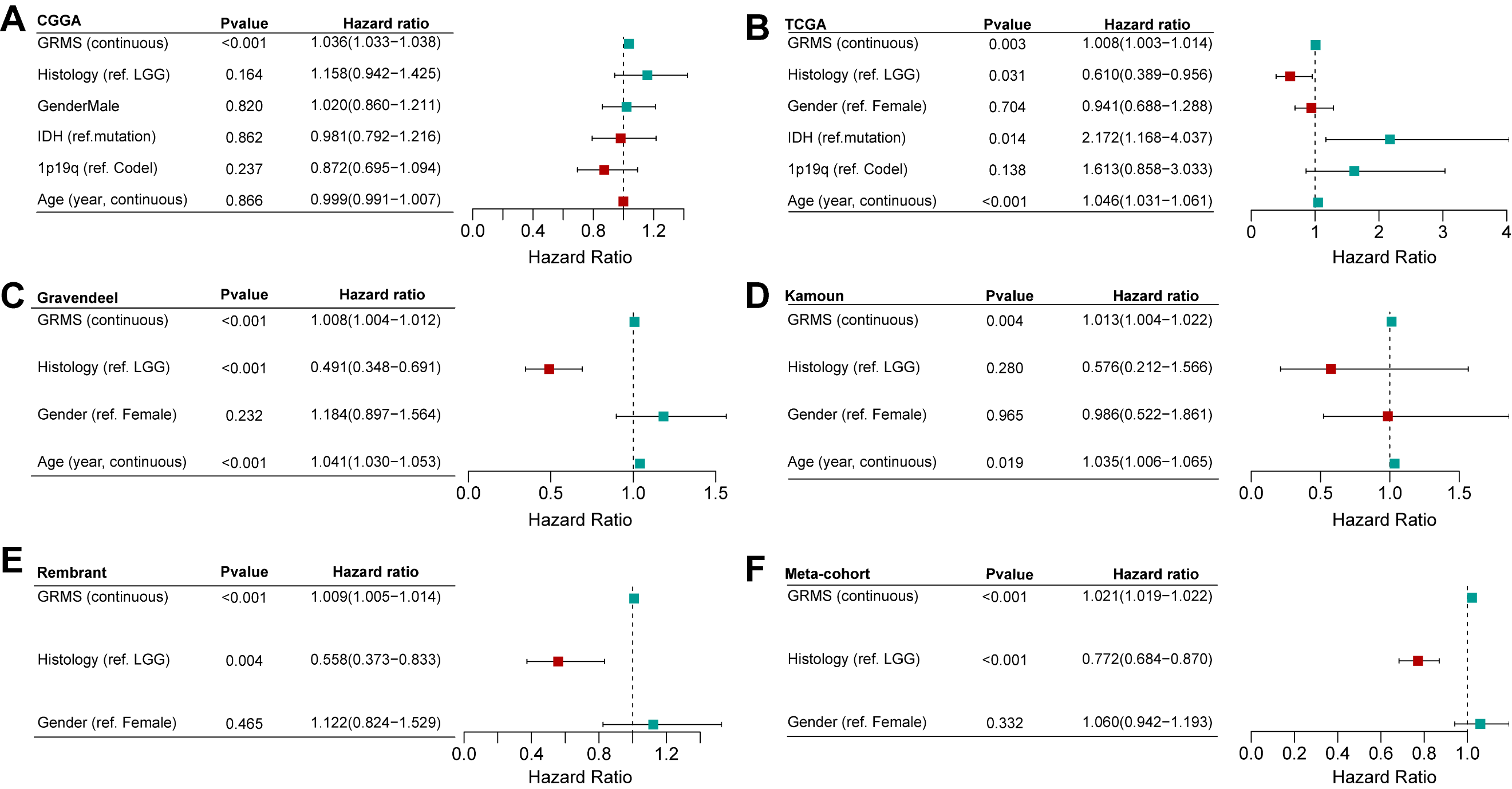


**Figure S5.** Multivariate Cox regression demonstrated that GRMS remained an independent prognostic risk factor (A-F).


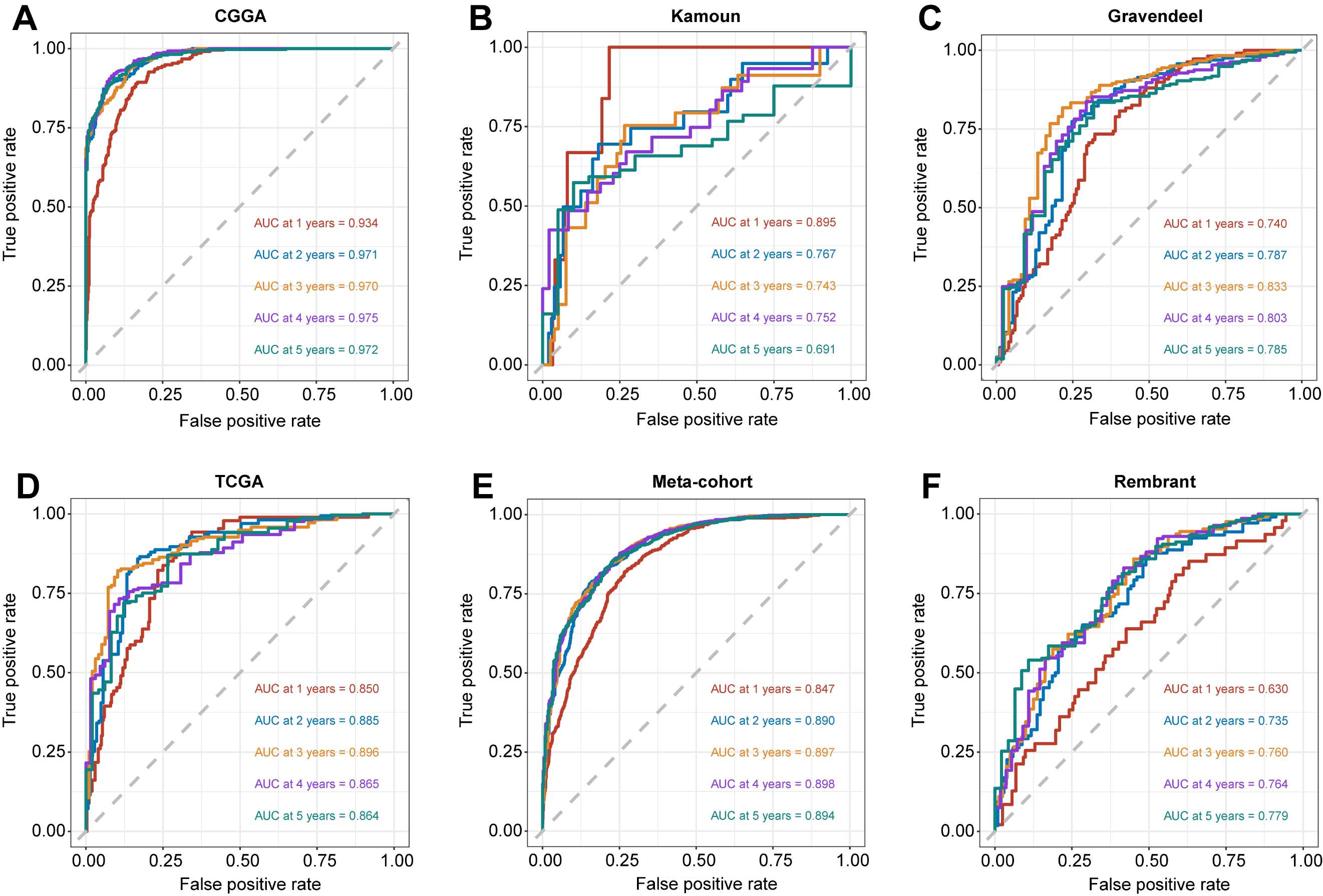


**Figure S6.** ROC analysis demonstrated the GRMS might be a strong indicator to predict glioma outcomes (A-F).


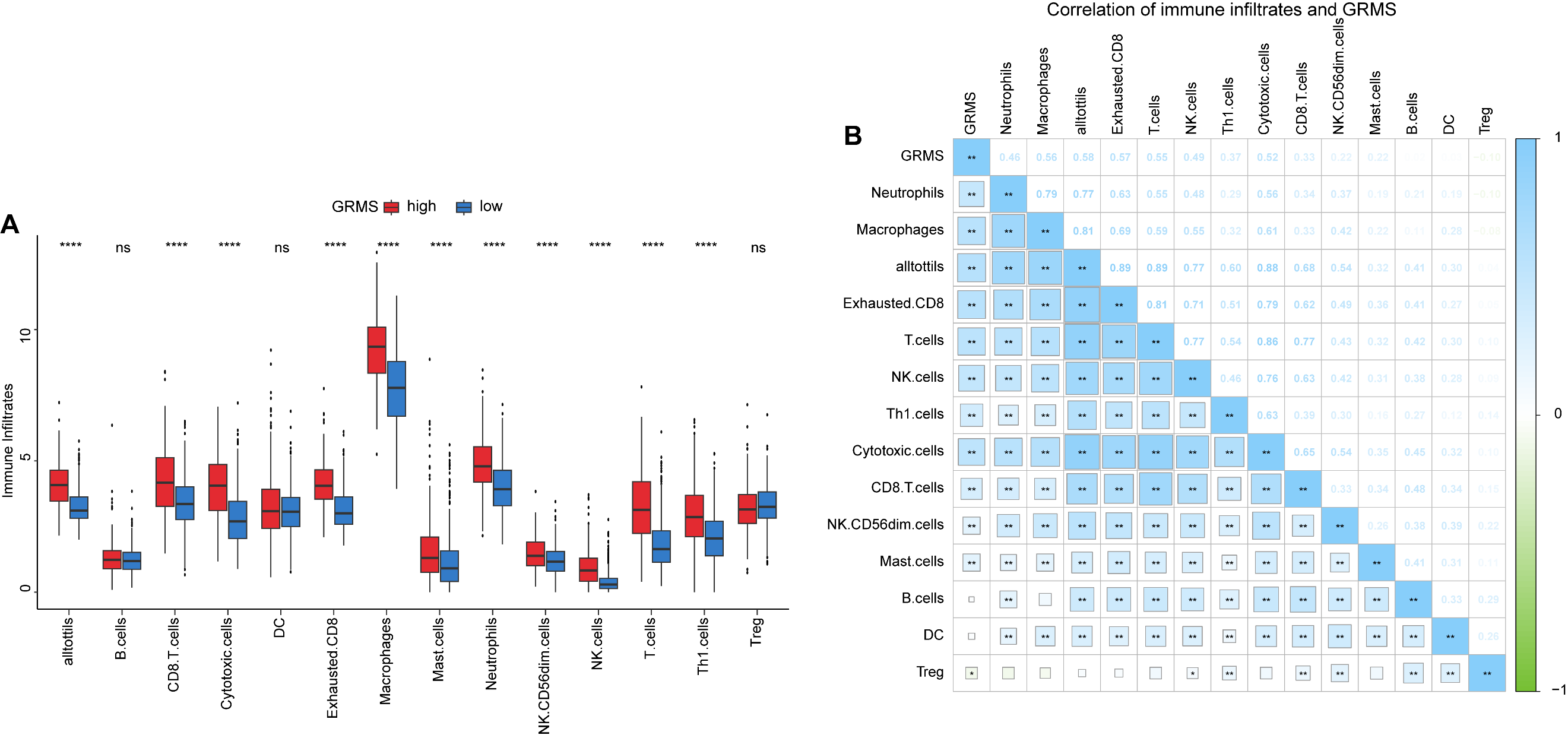


**Figure S7.** The boxplot demonstrated the patients in high GRMS group had more immune infiltrates(A). The heatmap demonstrated the correlation of GRMS value with immune infiltrates (B).


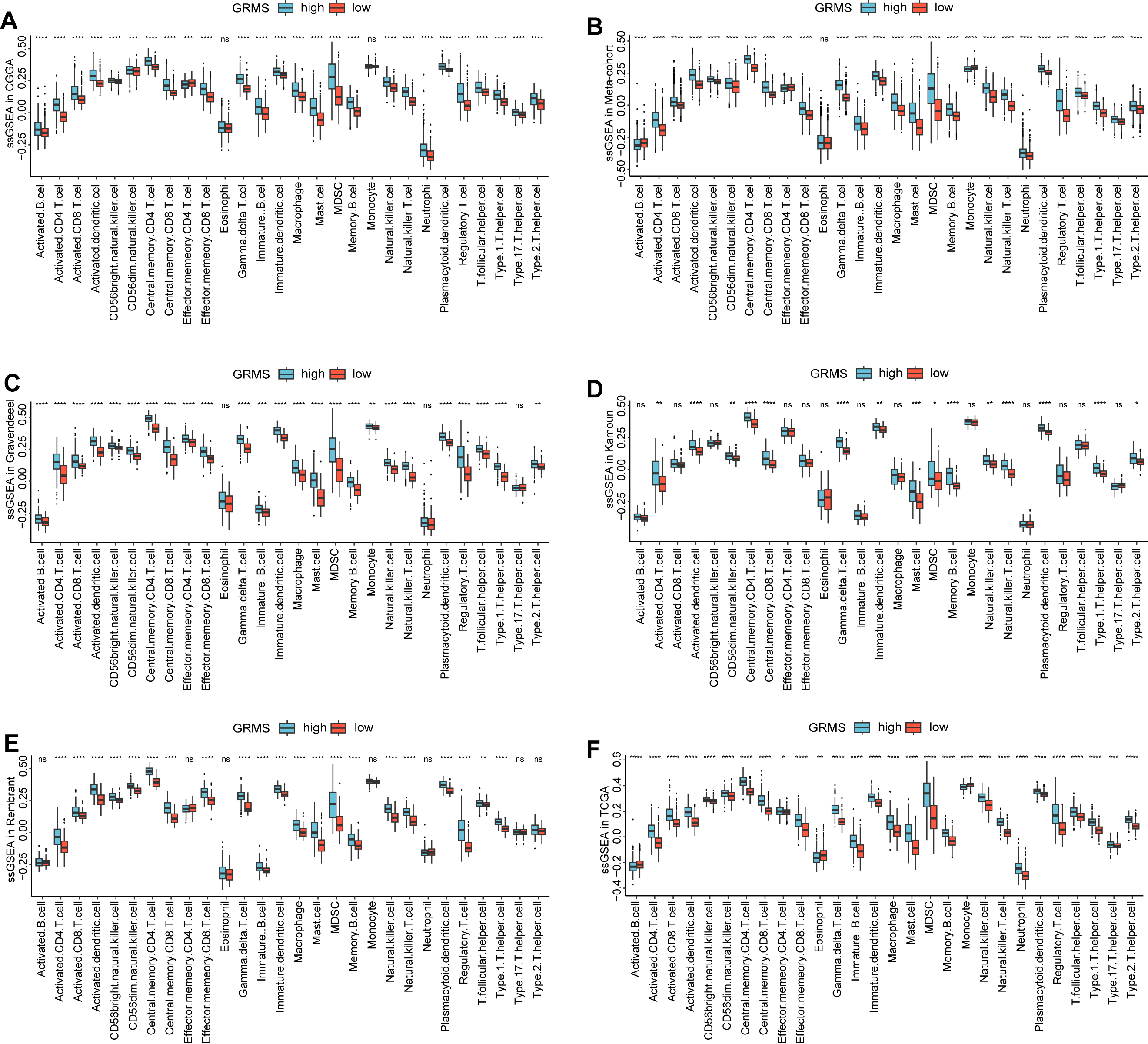


**Figure S8.** The boxplot demonstrated the patients in high GRMS group had more immune infiltrates(A-F).


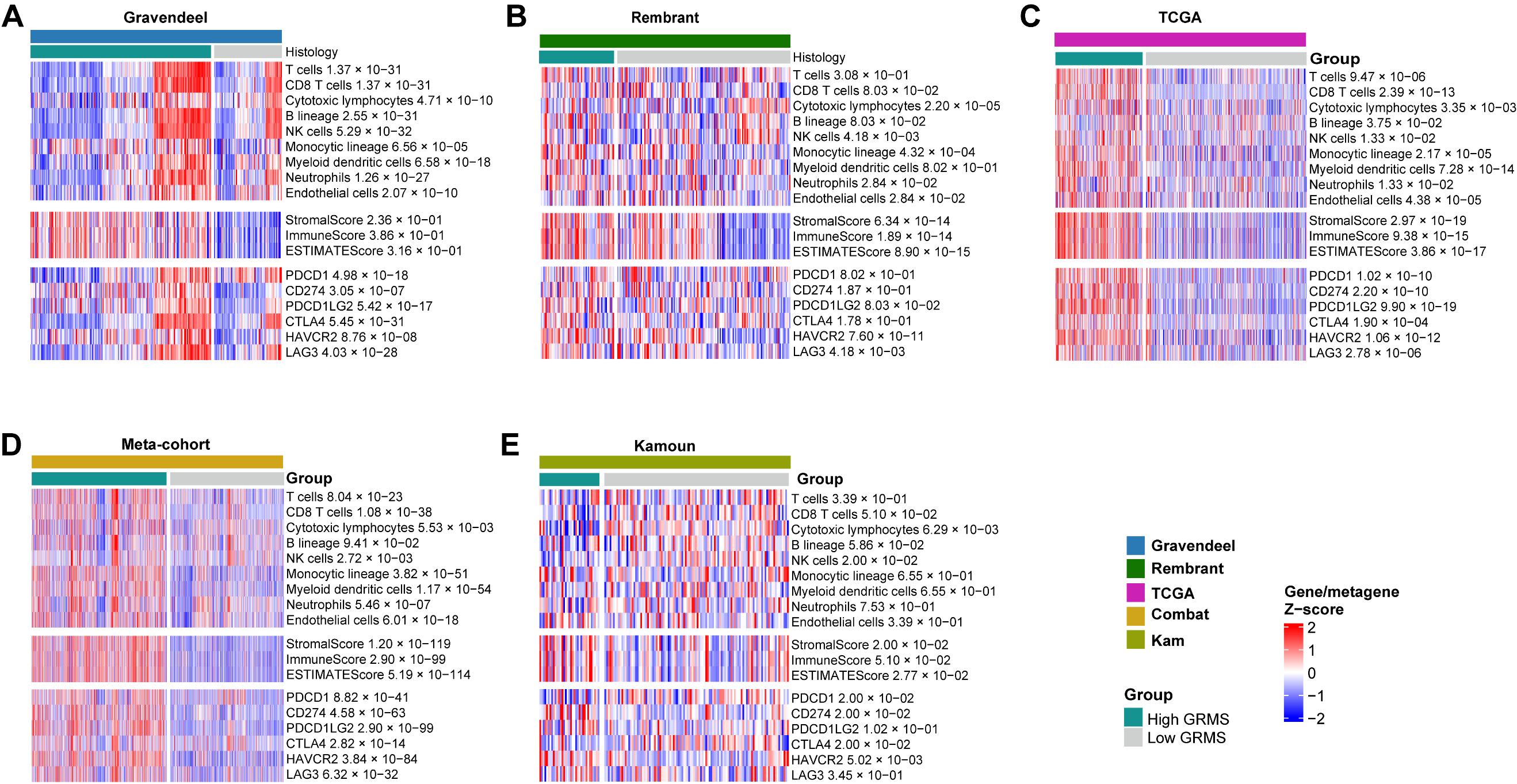


**Figure S9.** The heatmap demonstrated different immune landscape between GRMS subgroups (A-E).


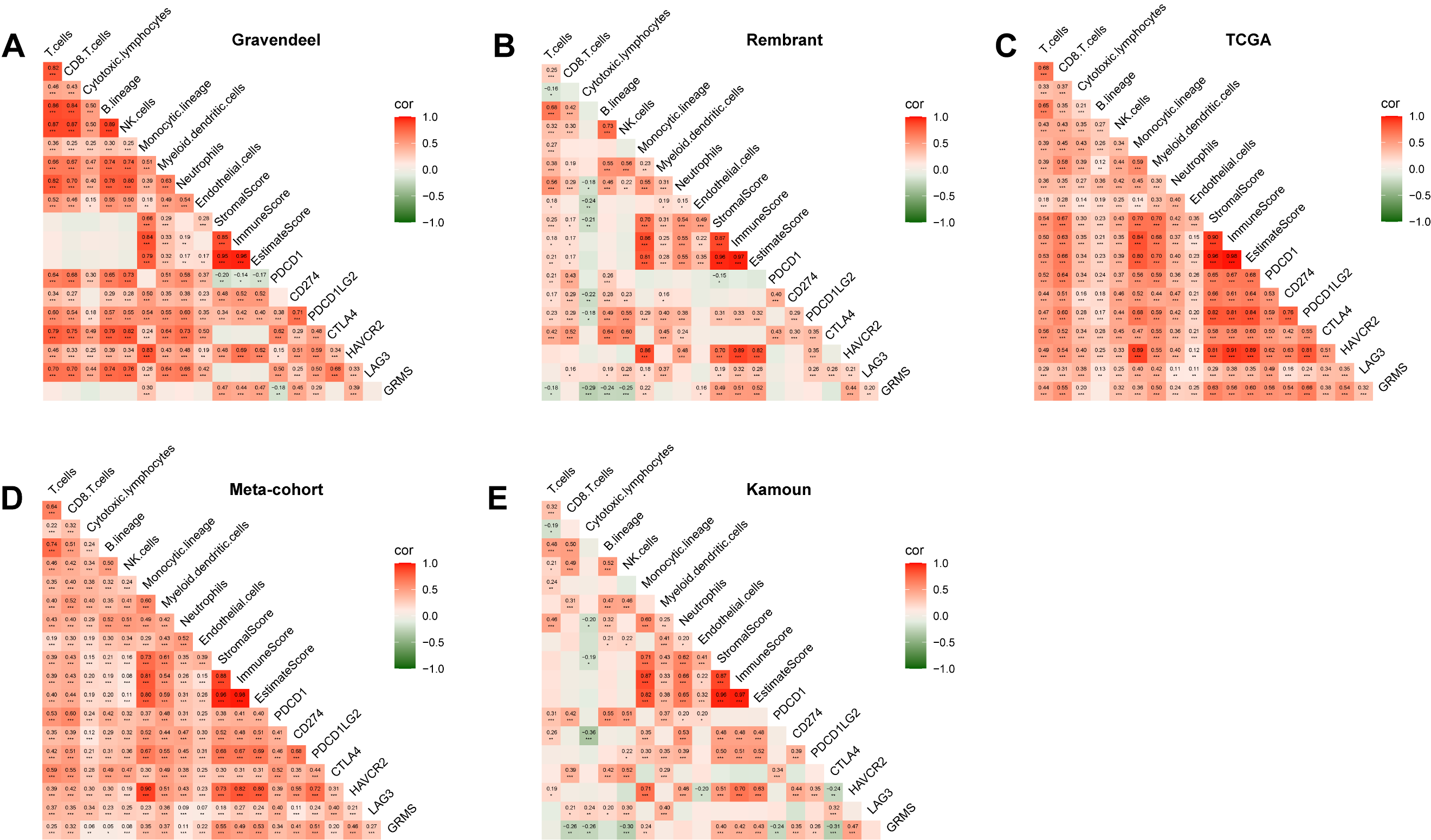


**Figure S10.** The heatmap demonstrated correlation levels between GRMS value and immune infiltrates and immune checkpoints (A-E).


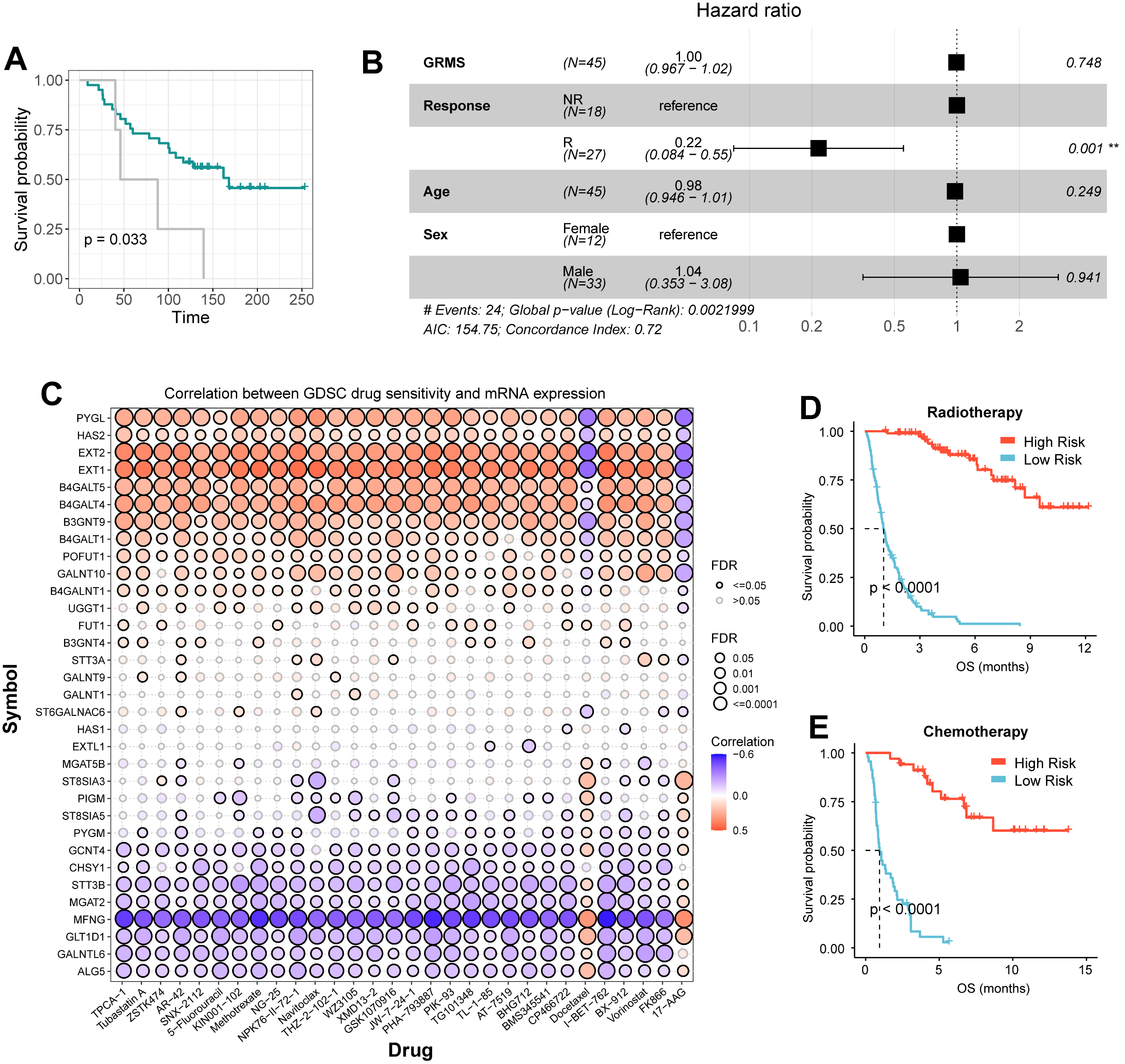


**Figure S11.** K-M curves of GRMS in predicting OS of patients who received immunotherapy (A). Multivariate Cox regression demonstrated that GRMS was not an independent prognostic risk factor for patients who received immunotherapy (B).

Correlation between glycosylation-related mRNAs and the clinical efficacy of brain cancer treatments based on GDSC database (C). K-M curves of survival advantage in patients treated with ART or ACT who had low GRMS (D-E).
